# Synthesis and evaluation of enantiomeric quinoline-2-carboxamides: positron emission tomography imaging agents for the translocator protein

**DOI:** 10.1039/d5md00930h

**Published:** 2025-12-11

**Authors:** Lachlan J. N. Waddell, Mark G. MacAskill, Holly McErlain, Timaeus E. F. Morgan, Lewis Williams, Victoria J. M. Reid, Anna Beyger, Sally L. Pimlott, Adriana A. S. Tavares, Andrew Sutherland

**Affiliations:** a School of Chemistry, University of Glasgow University Avenue Glasgow G12 8QQ UK Andrew.Sutherland@glasgow.ac.uk; b Edinburgh Imaging, University of Edinburgh 47 Little France Crescent Edinburgh EH16 4TJ UK; c University/BHF Centre for Cardiovascular Sciences, University of Edinburgh 47 Little France Crescent Edinburgh EH16 4TJ UK; d West of Scotland PET Centre, Greater Glasgow and Clyde NHS Trust Glasgow G12 OYN UK

## Abstract

The translocator protein (TSPO) is a key biomarker for inflammation. Positron emission tomography (PET) imaging of TSPO has emerged as a valuable tool for investigating multiple disorders throughout the body. We previously reported the development of [^18^F]LW223, a quinoline-2-carboxamide bearing an *R*-configured side chain, which binds TSPO independently of the rs6971 human polymorphism and enables quantification of macrophage-driven inflammation in the course of myocardial infarction. In the present study, we provide a comprehensive molecular and biological characterisation of LW223 and its *S*-enantiomer, further supporting their potential as PET imaging agents for human inflammatory processes. Two synthetic routes were developed: one enabling direct multigram-scale synthesis of LW223, and another allowing late-stage (radio)fluorination. The latter was applied for the synthesis of the *S*-enantiomer. Binding assays using homogenised human brain tissue revealed that the *S*-enantiomer exhibits 7.5-fold lower affinity (*K*_i_ = 4.5 ± 0.7 nM) than the *R*-enantiomer, yet remains insensitive to rs6971 polymorphism. Molecular docking studies with the X-ray structure of wild-type TSPO from *Bacillus cereus* provided insights into enantiomer-specific binding interactions. Collectively, these findings advance our understanding of LW223 as a TSPO-targeted PET ligand for human inflammatory disease.

## Abstract 2

Synthesis and biological testing of the *R*- and *S*-enantiomers of LW223, a third generation TSPO PET imaging agent using human brain tissue showed that despite a 7.5-fold difference in affinity, both are insensitive to rs6971 polymorphism.

## Graphical abstract

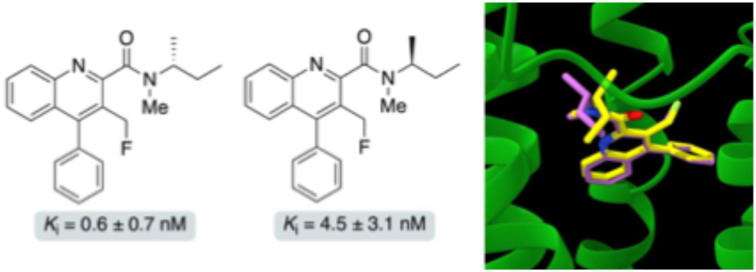

## Introduction

The translocator protein (TSPO), an 18 kDa mitochondrial membrane protein located on the outer mitochondrial membrane, has become an important biomarker for imaging neuroinflammation, cancer, and other diseases involving immune cell activation.^1,2^ This makes it a valuable target for positron emission tomography (PET), a non-invasive imaging modality able to quantify molecular processes *in vivo*.^3^ PET imaging of TSPO has been widely investigated in the context of neurodegenerative diseases such as Alzheimer's disease, Parkinson's disease, and multiple sclerosis, as well as in brain tumours and systemic inflammation.^4,5^

**Fig. 1:**
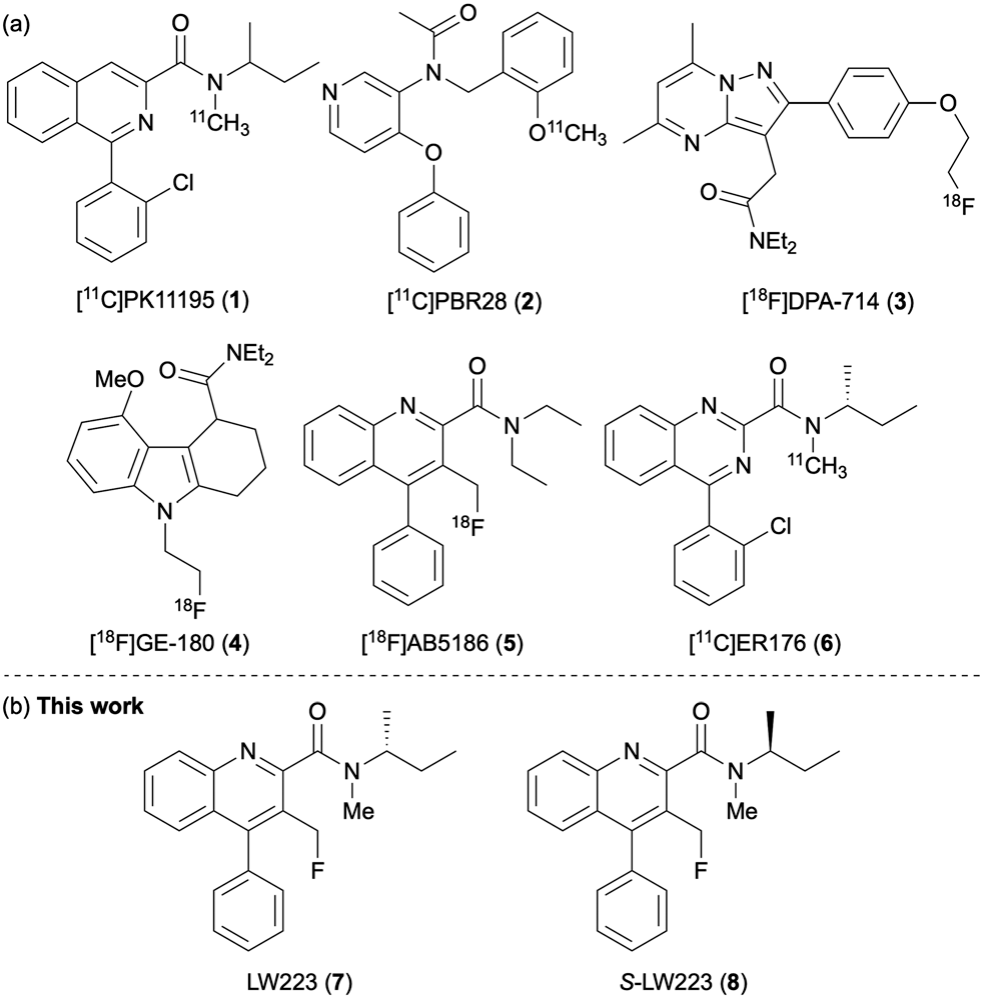
(a) PET imaging agents of TSPO. (b) This work: molecular and biological characterisation of LW223 enantiomers.

The first-generation TSPO PET tracer, [^11^C]PK11195 (1) (Fig. 1a), has been used for many years for the *in vivo* imaging of neuroinflammation but suffers from high nonspecific binding and poor signal-to-noise ratio.^4*a*,*b*,6,7^ In response, a series of second-generation radioligands, such as [^11^C]PBR28 (2),^8^ [^18^F]DPA-714 (3),^9^ [^18^F]GE-180 (4),^10^ and [^18^F]AB5186 (5)^5,11^ were developed to improve binding affinity, brain penetration, and overall image quality. While these agents represented significant progress, they revealed a critical challenge in human imaging: the influence of a common single-nucleotide polymorphism (rs6971) in the TSPO gene, which alters the binding affinity of many tracers.^12^ This polymorphism gives rise to three binding affinity phenotypes, high-affinity binders (HABs), mixed-affinity binders (MABs), and low-affinity binders (LABs), leading to significant variability in the PET signal that complicates both quantitative analysis and clinical interpretation.^13^ For example, some second-generation radioligands exhibit excellent binding properties in HABs but markedly reduced affinity in LABs, rendering them unsuitable for a significant portion of the population without genotyping.^12,14^ Even then, complex PET image analysis corrections are required to mitigate the effects of the phenotype. These limitations have driven the development of third-generation TSPO PET tracers designed to minimise or eliminate binding sensitivity to the rs6971 polymorphism.^15^ Promising candidates, such as [^11^C]ER176 (6), have shown reduced genotype-dependent variability while maintaining high affinity and favourable imaging characteristics.^16^

In 2021, we reported a new TSPO PET tracer, LW223 (7) (Fig. 1b).^17,18^*In vitro* assays showed that LW223 binding was unaffected by rs6971 in human brain and heart tissues, while *in vivo*, [^18^F]LW223 exhibited favourable pharmacokinetics, metabolic stability and dosimetry in rodents. PET imaging post-myocardial infarction (MI) revealed significantly increased TSPO signal in the infarcted myocardium, consistent with *ex vivo* macrophage markers. Subsequent studies using [^18^F]LW223 have led to improved protocols for quantifying TSPO expression in rat heart and brain,^19^ enabled high-sensitivity TSPO PET imaging in the murine brain^20^ and confirmed sexually dimorphic patterns of radiotracer uptake associated with TSPO.^21^ As the development of [^18^F]LW223 as a mainstream PET tracer for TSPO continues, we have sought to fully understand the molecular and biological characteristics of this imaging agent, including its insensitivity to rs6971 polymorphism and the role of the chiral amine side-chain in TSPO binding. Here, we report two synthetic routes that allow the large-scale preparation of LW223 or late-stage (radio)fluorination and access to [^18^F]LW223. We also describe the synthesis of the *S*-enantiomer (8) and, through binding assays with homogenised human brain tissue, demonstrate that although this compound exhibits 7.5-fold lower affinity, it remains insensitive to rs6971 polymorphism. Additionally, docking studies are presented that provide an insight of enantiomer-specific binding interactions.

## Results and discussion

### Synthetic chemistry

The design of LW223 (7) was based on our previously developed TSPO tracer, AB5186 (5), which despite its high affinity for TSPO (*K*_i_ = 2.8 nM), exhibited sensitivity to the rs6971 polymorphism.^11,17^ To overcome this limitation, we combined the quinoline-2-carboxamide core of AB5186 with the *sec*-butyl amine side-chain of PK11195 (1), proposing that the resulting structure would retain high TSPO affinity, while remaining insensitive to rs6971 polymorphism. As the *R*-enantiomer of PK11195 displays higher affinity for TSPO,^22^ this stereochemistry was initially adopted for LW223.

**Scheme 1:**
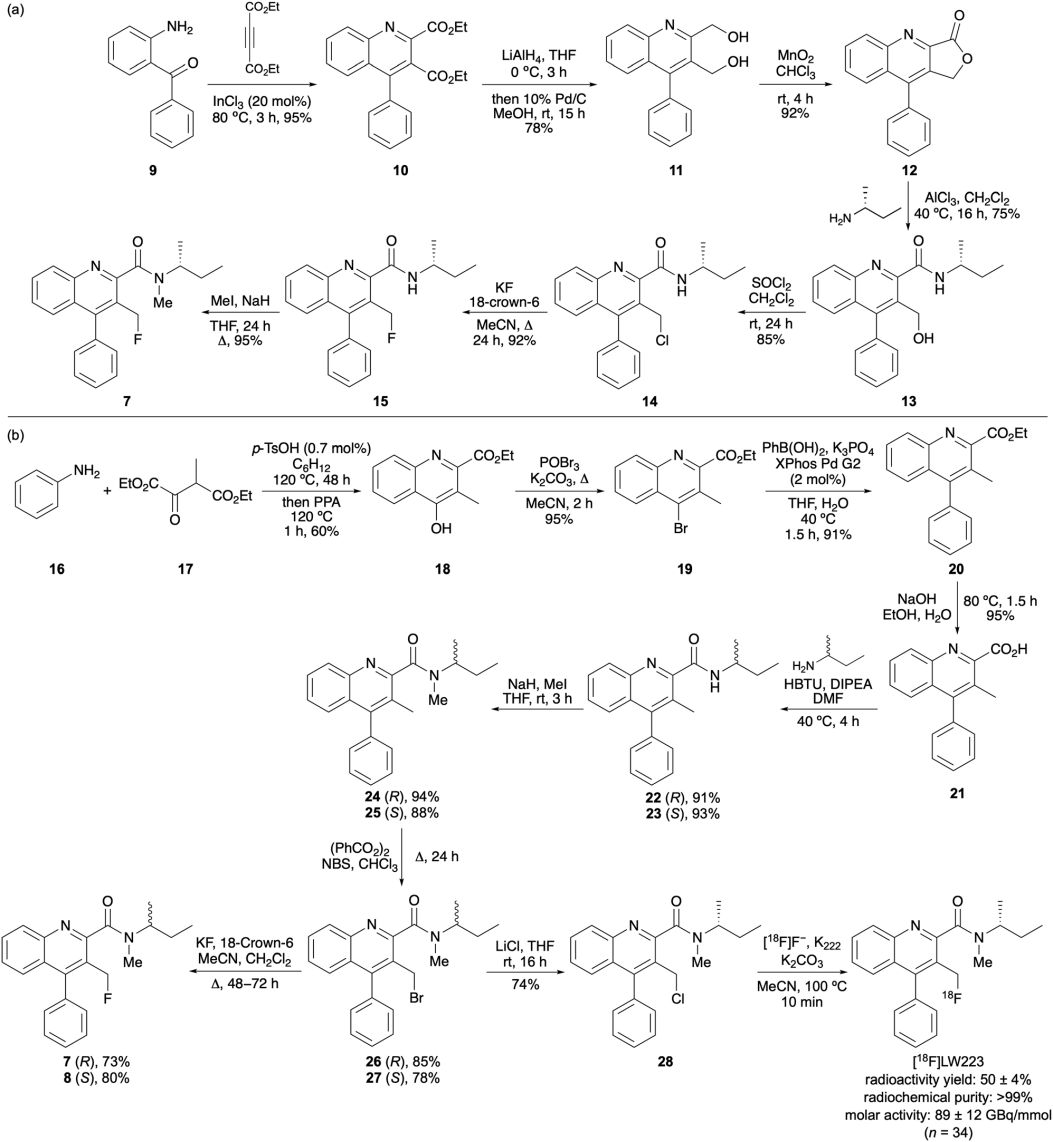
(a) Seven-step scalable synthesis of (*R*)-LW223 (7). (b) Second generation synthesis of LW223 that allows final-step (radio)fluorination.

The first key objective of the synthetic chemistry programme was the development of a scalable synthesis of LW223 that could generate material for extensive biological studies. Our previous synthesis of AB5186 required a six-step route to lactone 12 and included a ring-opening step using diethylamine and the highly pyrophoric Lewis acid, trimethylaluminium.^5^ In this study, we developed a more efficient three-step synthesis of lactone 12 and found an alternative to the use of pyrophoric Lewis acids (Scheme 1a). The quinoline-2-carboxamide core was accessed *via* a one-pot, two-component reaction between 2-aminobenzophenone (9) and diethyl acetylenedicarboxylate, catalysed by indium(iii) chloride, to afford 4-phenylquinoline 10 in quantitative yield.^23^ Direct reduction of the diester using lithium aluminium hydride, followed by rearomatisation using Pd/C gave the corresponding diol 11 in 78% yield.^24^ Conversion of the diol to lactone 12 using a manganese dioxide-mediated regioselective oxidation completed the three-step synthesis in 68% overall yield.

To eliminate the requirement for pyrophosphoric reagents, alternative aluminium(iii) Lewis acids were explored for the ring-opening of lactone 12. Aluminium(iii) chloride proved effective, and reaction with (*R*)-*sec*-butylamine gave the ring-opened product in 75% yield (Scheme 1a). Subsequent alcohol activation with thionyl chloride and then fluorination using potassium fluoride and 18-crown-6, followed by methylation of the amide under basic conditions completed the seven-step synthesis of LW223 (7) with a 38% overall yield. This optimised route enabled multigram-scale production of LW223.

The second key objective of the synthetic programme was to establish a route that would allow final-step fluorination, thereby facilitating radiofluorination and the production of [^18^F]LW223. The proposed strategy centred on the synthesis of a C-3 methyl quinoline-2-carboxamide, designed to undergo Wohl–Ziegler radical-initiated bromination,^25^ that would ultimately allow final-step (radio)fluorination (Scheme 1b). A 4-hydroxyquinoline intermediate was first prepared *via* the Combes reaction.^26^ Condensation of aniline with diethyl oxalpropionate (17) in the presence of catalytic *p*-tosic acid under Dean–Stark conditions afforded the corresponding imine. Subsequent acid-mediated cyclisation using polyphosphoric acid (PPA) gave 4-hydroxyquinoline 18 in 60% yield. Bromination with phosphorus oxybromide provided the corresponding 4-bromoquinoline 19, which was then subjected to a Suzuki–Miyaura cross-coupling reaction to install the 4-phenyl substituent. Previous syntheses of 4-aryl quinoline-2-carboxamides employed Pd(PPh_3_)_4_ (10 mol%) as the catalyst under conventional conditions and while this gave excellent yields, the reactions required high temperatures (120 °C) and long reaction times (16 h).^27^ In contrast, the use of the Buchwald pre-catalyst, XPhos Pd G2 enabled a more efficient reaction under milder conditions.^28^ Using only 2 mol% catalyst loading, coupling of 4-bromoquinoline 19 with phenylboronic acid at 40 °C was complete after 1.5 h and gave 20 in 91% yield.

Following ester hydrolysis with sodium hydroxide, various methods were explored for incorporation of the chiral amide side chain. Initial attempts involved conversion of the carboxylic acid to the corresponding acid chloride, followed by reaction with (*R*)-*sec*-butylamine. Although this approach gave the desired amide in 70% yield, it required super-stoichiometric amounts of the chiral amine (10–15 equiv.). A more effective method involved direct coupling of the carboxylic acid with (*R*)-*sec*-butylamine using HBTU and Hünig's base (Scheme 1b). This provided amide 22 in 91% yield using only 1.1 equivalents of (*R*)-*sec*-butylamine. Methylation with iodomethane and sodium hydride gave *N*-methyl amide 24 in quantitative yield. Finally, the C-4 methyl group was brominated and subsequently converted to the target fluoride. Wohl–Ziegler bromination^25^ using *N*-bromosuccinimide (NBS) and dibenzoyl peroxide afforded clean bromination at the C-4 methyl group and preparation of 26 in 85% yield. Substitution with potassium fluoride in the presence of 18-crown-6 completed another route to access LW223 (7). Analysis of LW223 (7) by chiral HPLC confirmed the retention of optical purity during the final steps of this route with a 99.5 : 0.5 er ratio (see SI). The main objective of this second synthetic route was to generate a precursor suitable for late-stage radiofluorination. To evaluate different halogen leaving groups, bromide 26 was converted to the corresponding chloride 28 by reaction with lithium chloride. Studies showed that chloride 28 exhibited greater stability than bromide 26, enabling long-term storage. Subsequent radiofluorination of 28 afforded cleaner reactions and easier purification. As previously reported by us, reaction of 28 with [^18^F]fluoride yields radiofluorinated LW223 (7) in 50% radioactivity yield and with excellent radiochemical purity.^29^

To access the *S*-enantiomer of LW223 for TSPO binding studies, this same route was utilised (Scheme 1b). The replacement of (*R*)-*sec*-butylamine with the *S*-enantiomer proceeded as expected and gave the corresponding products in similar yields.^30^ Overall, this approach allowed the synthesis of both enantiomers in 25–26% yields over the eight steps.

### Physicochemical analysis of LW223

The selection of potential candidates as neurological imaging agents requires evaluation of key physicochemical properties. To effectively cross brain capillary endothelial cells and penetrate the blood–brain barrier (BBB), a compound must exhibit sufficient plasma membrane permeability. For agents that rely primarily on passive diffusion, the membrane partition coefficient is particularly critical. Based on a previously reported study of HPLC methods for assessing these parameters,^31^ the partition coefficient (log *P*), membrane permeability (*P*_m_), membrane partition coefficient (*K*_m_) and plasma protein binding (% PPB) of LW223 (7), PK11195 (1) and AB5186 (5) were determined (Table 1). It should be noted that the original study performed by Tavares *et al.*,^31^ used ten successful imaging agents to establish the limits of each of these parameters (log *P* < 4, *P*_m_ < 0.5, *K*_m_ < 250, % PPB < 95%). With a membrane permeability of 0.56 and plasma protein binding of 92%, LW223 demonstrates physicochemical properties consistent with good BBB penetration. In addition, its membrane partition coefficient of 195 suggests a favourable ratio of specific to non-specific binding. Although the log *P* value of LW223 exceeds the upper limit, recent murine brain studies have confirmed that LW223 effectively crosses the BBB and exhibits low non-specific binding.^17,20^ Taken together, these physicochemical properties and *in vivo* studies support the potential of LW223 as a neurological imaging agent. Although the *R*-enantiomer of LW223 was used to determine the physicochemical properties in this study, these values are expected to be representative of the *S*-enantiomer, which is consistent with observations for other enantiomeric pairs.^32^

**Table 1:** Physicochemical properties of PK11195, AB5186 and LW223

Compound	log *P*^a^	*P*_m_ b	*K*_m_ b	% PPB^c^
PK11195 (1)	3.98	0.52	184	92%
AB5186 (5)	3.74	0.35	124	90%
LW223 (7)	4.13	0.56	195	92%

a Determined using C_18_ column.

b Determined using immobilised artificial membrane (IAM) column.

c Determined using human serum albumin (HAS) coated column.

### (*S*)-LW223 binding studies with TSPO in rs6971 genotyped human brain tissue

**Fig. 2:**
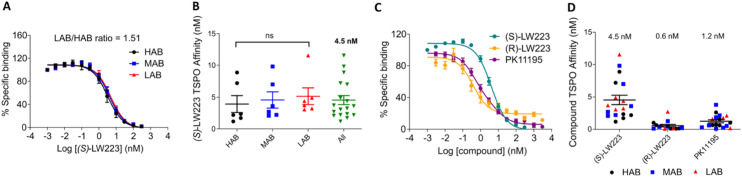
[^3^H]PK11195 competition binding assays on human brain tissue from HAB, MAB and LAB individuals. A) Average fitting binding curves for (*S*)-LW223 across the different groups, *n* = 6 for each genotype. B) Affinity values calculated from the individually fitted binding curves. ns = *P* > 0.05 for HAB *vs.* LAB using an unpaired *t*-test. C) Comparative binding curves for (*S*)-LW223, (*R*)-LW223 and PK11195 fitted across the average of all individual subjects. (*S*)-LW223 *n* = 18, (*R*)-LW223 *n* = 14 and PK11195 *n* = 18. D) Affinity values calculated from the individually fitted binding curves. The data for (*R*)-LW223 and PK11195 was generated as part of our previously published study (see ref. 17).

PET imaging with [^11^C]PK11195 (1) was originally conducted using a racemic mixture. However, subsequent findings demonstrated that the *R*-enantiomer exhibits approximately two-fold higher affinity for TSPO, leading to a general shift toward the use of the more active stereoisomer.^6,33^ After preparing both enantiomers of LW223, the next objective was to evaluate the impact of LW223 enantiomerism on TSPO binding affinity as well as sensitivity to the rs6971 polymorphism. Homogenised brain tissue samples were prepared from the frontal cortex of 18 genotyped human donors, comprising HABs, MABs and LABs, with *n* = 6 per group, and used in competition binding assays with [^3^H]PK11195 to assess the affinity of (*S*)-LW223 (8). These studies revealed that like (*R*)-LW223, the binding of (*S*)-LW223 is not significantly influenced by the rs6971 polymorphism (*p* = 0.5273; Fig. 2A and B). However, (*S*)-LW223 displayed a 7.5-fold lower affinity for TSPO compared to (*R*)-LW223 (4.5 ± 3.1 nM *vs.* 0.6 ± 0.7 nM, respectively; mean ± SD, Fig. 2C and D), and a 3.75-fold lower affinity relative to PK11195 (1.2 ± 1.0 nM). These findings indicate that although (*S*)-LW223 is not affected by the rs6971 polymorphism, the difference in binding affinity between the LW223 enantiomers is greater than that observed for stereoisomers of other TSPO ligands such as [^11^C]PK11195.^6,33^ Consequently, for TSPO PET imaging studies, (*R*)-LW223 rather than the *S*-enantiomer or the racemic mixture is preferable.

### Docking studies of (*R*)- and (*S*)-LW223 with *Bacillus cereus* TSPO

Given the insensitivity of the two LW223 enantiomers to TSPO polymorphism and the 7.5-fold difference in binding affinity, molecular docking studies were conducted to investigate their key interactions with the protein. Although crystal structures of TSPO from multiple species have been reported, including mutant variants that mimic human polymorphism, only a single structure to date has resolved the PK11195 binding site; the wild-type TSPO from *Bacillus cereus* (PDB code: 4RYI).^34,35^ As the LW223 enantiomers are direct structural analogues of PK11195, the *Bacillus cereus* TSPO crystal structure was selected for these studies.

**Fig. 3:**
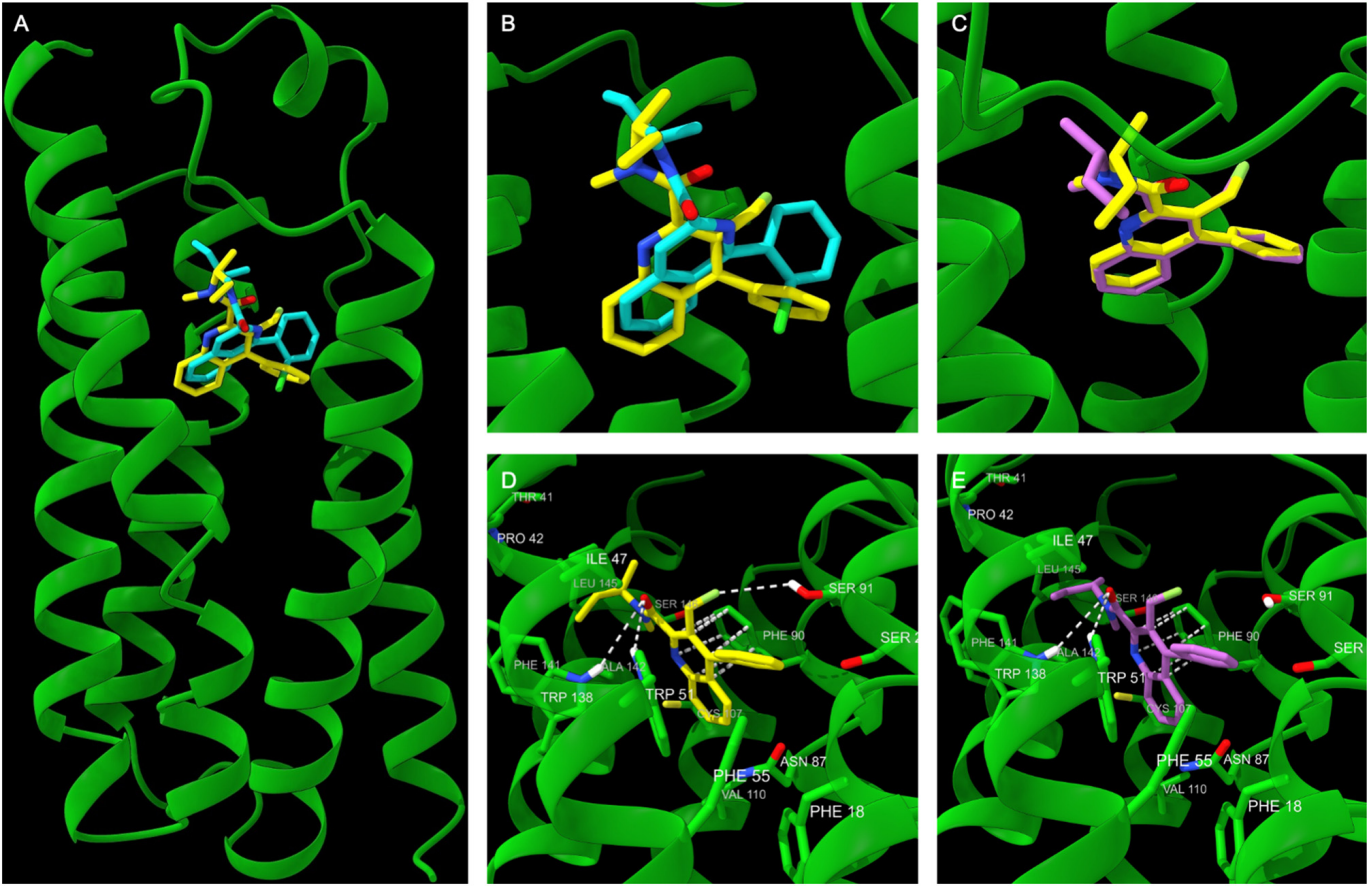
Docking studies with the X-ray structure of *Bacillus cereus* TSPO. A) Superimposed (*R*)-LW223 (yellow) and PK11195 (cyan). B) Close up view of superimposed (*R*)-LW223 (yellow) and PK11195 (cyan). C) Close up view of superimposed (*R*)-LW223 (yellow) and (*S*)-LW223 (violet). D) Close up view of docked (*R*)-LW223 showing key interactions. E) Close up view of docked (*S*)-LW223 showing key interactions.

Docking studies within the PK11195 binding site showed that the quinoline and isoquinoline cores of (*R*)-LW223 and PK11195 occupy similar spatial positions, whereas their twisted 4-phenyl rings and amide side chains engage distinct lipophilic sub-pockets (Fig. 3A and B). Although the carbonyl groups also adopt different orientations, the same key hydrogen bonds to Trp51 and Trp138 are maintained. Crucially, (*R*)-LW223, like PK11195 avoids close contact with Ala142, the residue corresponding to the Ala147 → Thr147 polymorphism in human TSPO.^34,35^ This lack of interaction could explain the polymorphism-insensitive binding profile of (*R*)-LW223.

General comparison of the docked (*R*)- and (*S*)-LW223 enantiomers revealed similar positioning of the twisted 4-phenylquinoline cores with π–π stacking interactions to Phe90, but as expected, notable differences were observed in the orientation of the amide side chains (Fig. 3C). Detailed analysis showed that for (*R*)-LW223, the amide carbonyl forms hydrogen bonds with Trp138 and Trp51 with distances of 4.1 Å and 3.2 Å, respectively (Fig. 3D), compared to 4.2 Å and 3.3 Å for the *S*-enantiomer (Fig. 3E). Additionally, the fluorine–Ser91 interaction was also more favourable for the *R*-enantiomer (3.7 Å *vs.* 4.3 Å). The amide side chains of both enantiomers are positioned within a hydrophobic pocket, forming van der Waals contacts with residues such as Pro42, Ile47, Leu145, and Phe141; however, these contacts are generally shorter for the *R*-enantiomer suggesting a tighter fit. Notably, the amide of the *R*-enantiomer adopts a lower energy *Z*-conformation, compared to the *S*-enantiomer, in which the orientation of the carbonyl and *sec*-butyl group is more twisted. Although docking studies provide only qualitative guidance, these results offer some structural rationale for the higher TSPO affinity of (*R*)-LW223 compared to the (*S*)-enantiomer. In a similar manner to (*R*)-LW223, the *S*-enantiomer also avoids close contact with Ala142. Again, this possibly explains the exhibited insensitivity of (*S*)-LW223 to rs6971 polymorphism.

## Conclusions

In summary, two robust synthetic routes have been established for the TSPO ligand LW223: a multigram-scale synthesis and a late-stage (radio)fluorination strategy, the latter also enabling access to the *S*-enantiomer for investigation of enantioselective binding with TSPO. Binding studies with human brain tissue homogenates revealed that the *S*-enantiomer, while exhibiting 7.5-fold lower affinity than the *R*-enantiomer remains insensitive to the rs6971 polymorphism. Molecular docking with the *Bacillus cereus* TSPO structure provided some structural insight into the enantioselective binding profile of LW223 and the insensitivity of these compounds to rs6971 human polymorphism. These findings support the continued development of (*R*)-LW223 as a TSPO-targeted PET ligand for imaging human inflammatory processes and highlight its suitability for further translational investigation.

## Experimental

### General experimental

All reagents and starting materials were obtained from commercial sources and used as received. *N*-Bromosuccinimide was recrystallised from water and dried under high vacuum before use. Dry solvents were purified using a PureSolv 500 MD solvent purification system. All reactions were performed open to air unless otherwise mentioned. Brine refers to a saturated solution of sodium chloride. Flash column chromatography was performed using Merck Millipore matrix silica gel 60 (40–63 μm). Merck aluminium-backed plates pre-coated with silica gel 60 (UV_254_) were used for thin-layer chromatography and visualised with a UV lamp. ^1^H NMR and ^13^C NMR spectra were recorded on a Bruker DPX 400 spectrometer, with chemical shift values reported in ppm relative to tetramethylsilane (*δ*_H_ 0.00 and *δ*_C_ 0.00), or residual chloroform (*δ*_H_ 7.26), dimethylsulfoxide (*δ*_H_ 2.50) or methanol (*δ*_H_ 3.31) as standard. For ^13^C NMR the chemical shifts are reported relative to the central resonance of CDCl_3_ (*δ*_C_ 77.2) or CD_3_OD (*δ*_C_ 49.0) as standard. Proton and carbon assignments are based on two-dimensional COSY, HSQC, HMBC and DEPT experiments. Mass spectra were obtained using a Bruker Microtof-q for ESI, Agilent 6125B for CI, and JEOL JMS-700 for EI. Infrared spectra were obtained neat using a Shimadzu IR Prestige-21 spectrometer or Shimadzu 8400S spectrometer; wavenumbers are indicated in cm^−1^. Melting points were determined on either a Reichert platform melting point apparatus or Stuart Scientific melting point apparatus. Optical rotations were determined as solutions irradiating with the sodium D line (*λ* = 589 nm) using an Autopol V polarimeter. [*α*]_D_ values are given in units 10^−1^ deg cm^2^ g^−1^.

#### Diethyl 4-phenylquinoline-2,3-dicarboxylate (10)^23^

Indium(iii) chloride (0.680 g, 3.07 mmol) was added to 2-aminobenzophenone (9) (5.00 g, 15.3 mmol) and diethyl acetylenedicarboxylate (2.95 mL, 18.4 mmol) and the neat mixture stirred at 80 °C for 3 h. On cooling to ambient temperature, water (50 mL) was added and the mixture extracted using ethyl acetate (150 mL). The organic layer was then washed with water (100 mL) and brine (100 mL), dried (MgSO_4_), filtered and concentrated *in vacuo*. Purification by flash column chromatography (15% ethyl acetate in hexane) gave diethyl 4-phenylquinoline-2,3-dicarboxylate (10) (5.09 g, 95%) as a yellow solid. Mp 89–91 °C, lit.^23^ 95–96 °C; ^1^H NMR (400 MHz, CDCl_3_) *δ* 0.99 (3H, t, *J* = 7.2 Hz, OCH_2_*CH*_*3*_), 1.47 (3H, t, *J* = 7.2 Hz, OCH_2_*CH*_*3*_), 4.09 (2H, q, *J* = 7.2 Hz, O*CH*_*2*_CH_3_), 4.54 (2H, q, *J* = 7.2 Hz, O*CH*_*2*_CH_3_), 7.34–7.40 (2H, m, 2 × ArH), 7.47–7.53 (3H, m, 3 × ArH), 7.57 (1H, ddd, *J* = 8.4, 6.4, 1.6 Hz, ArH), 7.63 (1H, ddd, *J* = 8.4, 1.6, 0.6 Hz, ArH), 7.81 (1H, ddd, *J* = 8.4, 6.4, 1.6 Hz, ArH), 8.38 (1H, ddd, *J* = 8.4, 1.6, 0.6 Hz, ArH); ^13^C{^1^H} NMR (101 MHz, CDCl_3_) *δ* 13.8 (CH_3_), 14.4 (CH_3_), 61.7 (CH_2_), 62.8 (CH_2_), 126.8 (CH), 127.2 (C), 127.7 (C), 128.4 (2 × CH), 128.9 (CH), 129.2 (CH), 129.6 (2 × CH), 130.8 (CH), 131.1 (CH), 134.9 (C), 146.0 (C), 147.3 (C), 148.1 (C), 165.5 (C), 167.3 (C); MS (EI) *m*/*z* 349 (M^+^, 60%), 305 (20), 276 (55), 205 (100), 204 (82), 165 (12), 84 (5); HRMS (EI) *m*/*z*: [M]^+^ calcd for C_21_H_19_NO_4_ 349.1314; found 349.1311.

#### 9-Phenylfuro[3,4-*b*]quinolin-3(1*H*)-one (12)^24^

A suspension of lithium aluminium hydride (3.90 g, 103 mmol) in anhydrous diethyl ether (90 mL) was cooled to 0 °C, and to this was added dropwise, a solution of diethyl 4-phenylquinoline-2,3-dicarboxylate (10) (9.00 g, 26.0 mmol) in anhydrous diethyl ether (90 mL) under argon. The temperature of the reaction mixture was maintained at 0 °C and stirred vigorously for 3 h. After gradually warming to ambient temperature, the mixture was quenched by the dropwise addition of a 10% aqueous solution of potassium sodium tartrate (100 mL) and stirred for 4 h. The resultant mixture was extracted with diethyl ether (3 × 100 mL) and the combined organic extracts were dried (Na_2_SO_4_), filtered and concentrated *in vacuo* to afford a colourless residue which was dissolved in methanol (250 mL). 10% palladium on carbon (0.90 g) was added to the reaction flask and the resultant suspension stirred at ambient temperature for 15 h. The suspension was then filtered through a pad of Celite®, washed with methanol and concentrated *in vacuo*. Purification by trituration with diethyl ether afforded 2,3-bis(hydroxymethyl)-4-phenylquinoline (11) as a white solid (5.40 g, 78%). Diol 11 was taken onto the next step without any further purification. To a solution of 2,3-bis(hydroxymethyl)-4-phenylquinoline (11) (5.00 g, 18.9 mmol) in chloroform (300 mL) was added activated manganese(iv) oxide (32.9 g, 378 mmol) in several portions. The resultant suspension was stirred vigorously at ambient temperature for 4 h and then filtered through Celite®. After washing with chloroform (500 mL), the solvent was removed *in vacuo* to afford 9-phenylfuro[3,4-*b*]quinolin-3(1*H*)-one (12) as a white solid (4.53 g, 92%), which was used without further purification. Mp 188–190 °C, lit.^24^ 190–192 °C; ^1^H NMR (400 MHz, CDCl_3_) *δ* 5.40 (2H, s, OCH_2_), 7.45 (2H, dd, *J* = 8.0, 2.0 Hz, 2 × ArH), 7.57–7.70 (4H, m, 4 × ArH), 7.84–7.90 (1H, m, ArH), 7.91 (1H, d, *J* = 8.4 Hz, ArH), 8.43 (1H, d, *J* = 8.4 Hz, ArH); ^13^C{^1^H} NMR (101 MHz, CDCl_3_) *δ* 67.9 (CH_2_), 125.9 (CH), 128.0 (C), 129.0 (2 × CH), 129.45 (2 × CH), 129.51 (CH), 129.7 (CH), 130.8 (CH), 131.5 (CH), 132.4 (C), 133.7 (C), 144.0 (C), 144.4 (C), 150.8 (C), 168.8 (C); MS (ESI) *m*/*z* 284 (M + Na^+^, 100%); HRMS (ESI) *m*/*z*: [M + Na]^+^ calcd for C_17_H_11_NNaO_2_ 284.0682; found 284.0677.

#### (*R*)-*N*-(*sec*-Butyl)-3-(hydroxymethyl)-4-phenylquinoline-2-carboxamide (13)

To a stirred suspension of aluminium trichloride (4.0 g, 30 mmol) in anhydrous dichloromethane (130 mL) was added (*R*)-*sec*-butylamine (3.0 mL, 30 mmol) dropwise under argon. The resultant mixture was stirred for 0.5 h, before addition of a solution of 9-phenylfuro[3,4-*b*]quinolin-3(1*H*)-one (12) (2.6 g, 10 mmol) in dichloromethane (130 mL). The mixture was warmed at 40 °C for 16 h, before quenching by addition of and aqueous solution of 2 M hydrochloric acid (150 mL). The aqueous phase was extracted with dichloromethane (3 × 200 mL) and the combined organic extracts were dried (Na_2_SO_4_), filtered and concentrated *in vacuo*. Purification by flash column chromatography (10% ethyl acetate in petroleum ether) gave (*R*)-*N*-(*sec*-butyl)-3-(hydroxymethyl)-4-phenylquinoline-2-carboxamide (13) as a white solid (2.6 g, 75%). Mp 133–134 °C; IR (neat) 3387, 2961, 1651, 1518, 1402, 1161, 1015 cm^−1^; [*α*]_D_^30^ −24.8 (*c* 1.0, CHCl_3_); ^1^H NMR (400 MHz, CDCl_3_) *δ* 1.04 (3H, t, *J* = 7.5 Hz, CH_2_C*H*_3_), 1.35 (3H, d, *J* = 6.6 Hz, CHC*H*_3_), 1.65–1.76 (2H, m, C*H*_*2*_CH_3_), 4.11–4.22 (1H, m, CH), 4.67 (2H, d, *J* = 7.6 Hz, C*H*_2_OH), 5.40 (1H, t, *J* = 7.6 Hz, OH), 7.34–7.38 (2H, m, 2 × ArH), 7.47–7.56 (5H, m, 5 × ArH), 7.69–7.76 (1H, m, ArH), 8.13 (1H, dt, *J* = 8.4, 0.9 Hz, ArH), 8.28 (1H, d, *J* = 8.4 Hz, NH); ^13^C{^1^H} NMR (101 MHz, CDCl_3_) *δ* 10.7 (CH_3_), 20.5 (CH_3_), 29.9 (CH_2_), 47.3 (CH), 59.9 (CH_2_), 127.2 (CH), 128.2 (CH), 128.4 (CH), 128.6 (CH), 128.6 (CH), 128.7 (C), 129.7 (CH), 129.9 (CH), 130.0 (CH), 130.0 (CH), 131.5 (C), 136.0 (C), 145.5 (C), 149.8 (C), 150.1 (C), 166.4 (C); MS (ESI) *m*/*z* 357 (M + Na^+^, 100%); HRMS (ESI) *m*/*z*: [M + Na]^+^ calcd for C_21_H_22_N_2_NaO_2_ 357.1573; found 357.1563.

#### (*R*)-*N*-(*sec*-Butyl)-3-(chloromethyl)-4-phenylquinoline-2-carboxamide (14)

To a solution of (*R*)-*N*-(*sec*-butyl)-3-(hydroxymethyl)-4-phenylquinoline-2-carboxamide (13) (2.4 g, 6.7 mmol) in anhydrous dichloromethane (200 mL) was added thionyl chloride (10 mL, 140 mmol) under argon. The resultant solution was stirred at room temperature for 24 h. The reaction mixture was concentrated *in vacuo* and subsequently azeotroped with toluene to yield an off-white solid. Purification by trituration (diethyl ether) yielded (*R*)-*N*-(*sec*-butyl)-3-(chloromethyl)-4-phenylquinoline-2-carboxamide (14) as a white solid (2.0 g, 85%). Mp 218–219 °C; IR (neat) 3277, 2965, 1651, 1637, 1541, 1277, 1163 cm^−1^; [*α*]_D_^30^ −28.3 (*c* 1.0, CHCl_3_); ^1^H NMR (400 MHz, CDCl_3_) *δ* 1.04 (3H, t, *J* = 7.4 Hz, CH_2_C*H*_3_), 1.35 (3H, d, *J* = 6.6 Hz, CHC*H*_3_), 1.62–1.77 (2H, m, C*H*_2_CH_3_), 4.14–4.20 (1H, m, CH), 5.18 (1H, d, *J* = 10.4 Hz, C*H*HCl), 5.24 (1H, d, *J* = 10.4 Hz, CH*H*Cl), 7.33–7.41 (3H, m, 3 × ArH), 7.46–7.52 (1H, m, ArH), 7.53–7.60 (3H, m, 3 × ArH), 7.73 (1H, t, *J* = 8.1 Hz, ArH), 7.88 (1H, d, *J* = 8.1 Hz, NH), 8.17 (1H, d, *J* = 8.4 Hz, ArH); ^13^C{^1^H} NMR (101 MHz, CDCl_3_) *δ* 10.7 (CH_3_), 20.5 (CH_3_), 30.0 (CH_2_), 40.7 (CH_2_), 47.1 (CH), 127.1 (CH), 128.2 (CH), 128.4 (C), 128.6 (C), 128.7 (2 × CH), 128.8 (CH), 129.51 (CH), 129.53 (CH), 129.8 (CH), 130.4 (CH), 135.2 (C), 145.8 (C), 149.2 (C), 151.0 (C), 165.3 (C); MS (ESI) *m*/*z* 375 (M + Na^+^, 100%); HRMS (ESI) *m*/*z*: [M + Na]^+^ calcd for C_21_H_21_^35^ClN_2_NaO 375.1235; found 375.1220.

#### (*R*)-*N*-(*sec*-Butyl)-3-(fluoromethyl)-4-phenylquinoline-2-carboxamide (15)

To a solution of 18-crown-6 (1.4 g, 5.2 mmol) in anhydrous acetonitrile (100 mL) was added potassium fluoride (0.90 g, 16 mmol) and the resultant suspension stirred at ambient temperature for 0.5 h under argon. A solution of (*R*)-*N*-(*sec*-butyl)-3-(chloromethyl)-4-phenylquinoline-2-carboxamide (14) (1.8 g, 5.2 mmol) in anhydrous acetonitrile:dichloromethane (2 : 1, 150 mL) was then added dropwise to the reaction mixture and stirred under reflux for 24 h. On cooling to ambient temperature, water (100 mL) was added to the mixture and then extracted with dichloromethane (3 × 200 mL). The combined organic extracts were dried (Na_2_SO_4_), filtered and concentrated *in vacuo*. Purification by flash column chromatography (30% ethyl acetate in petroleum ether) gave (*R*)-*N*-(*sec*-butyl)-3-(fluoromethyl)-4-phenylquinoline-2-carboxamide (15) as a white solid (1.6 g, 92%). Mp 176–177 °C; IR (neat) 3283, 2965, 1640, 1539, 1452, 1402, 1207, 1157, 980 cm^−1^; [*α*]_D_^30^ −25.5 (*c* 0.5, CHCl_3_); ^1^H NMR (400 MHz, CDCl_3_) *δ* 1.04 (3H, t, *J* = 7.4 Hz, CH_2_C*H*_3_), 1.34 (3H, d, *J* = 6.6 Hz, CHC*H*_3_), 1.62–1.78 (2H, m, C*H*_2_CH_3_), 4.11–4.24 (1H, m, C*H*CH_3_), 5.79 (1H, dd, *J* = 11.4, 9.8 Hz, C*H*HF), 5.91, (1H, dd, *J* = 11.4, 9.8 Hz, CH*H*F), 7.29–7.36 (2H, m, 2 × ArH), 7.45–7.57 (5H, m, 5 × ArH), 7.74–7.79 (1H, m, ArH), 7.90 (1H, d, *J* = 8.1 Hz, NH), 8.16 (1H, d, *J* = 8.5 Hz, ArH); ^13^C{^1^H} NMR (101 MHz, CDCl_3_) *δ* 10.6 (CH_3_), 20.5 (CH_3_), 29.9 (CH_2_), 47.0 (CH), 78.6 (CH_2_, ^1^*J*_C–F_ = 162.9 Hz), 125.7 (C, ^2^*J*_C–F_ = 15.1 Hz), 127.2 (CH), 128.2 (CH), 128.4 (C, ^4^*J*_C–F_ = 1.9 Hz), 128.5 (CH), 128.7 (CH), 129.30 (CH), 129.84 (CH), 130.6 (CH, ^5^*J*_C–F_ = 0.9 Hz), 135.3 (C), 146.3 (C, ^3^*J*_C–F_ = 2.4 Hz), 150.1 (C), 152.6 (C, ^3^*J*_C–F_ = 4.9 Hz), 165.2 (C); MS (ESI) *m*/*z* 359 (M + Na^+^, 100%); HRMS (ESI) *m*/*z*: [M + Na]^+^ calcd for C_21_H_21_FN_2_NaO 359.1530; found 359.1514.

#### (*R*)-*N*-(*sec*-Butyl)-3-(fluoromethyl)-*N*-methyl-4-phenylquinoline-2-carboxamide (LW223, 7)

To a solution of (*R*)-*N*-(*sec*-butyl)-3-(fluoromethyl)-4-phenylquinoline-2-carboxamide (15) (1.5 g, 4.5 mmol) in anhydrous tetrahydrofuran (90 mL) was added sodium hydride (60% dispersion in mineral oil, 0.36 g, 9.0 mmol) under argon. The mixture was stirred at room temperature for 0.1 h, before the addition of iodomethane (1.4 mL, 23 mmol). The resultant solution was stirred under reflux for 24 h, before being quenching by addition of water (50 mL). The aqueous phase was extracted with diethyl ether (3 × 100 mL). The combined organic phase was washed with a 10% aqueous solution of sodium thiosulfate (100 mL) and brine (100 mL), dried (Na_2_SO_4_), filtered and concentrated *in vacuo*. Purification by column chromatography (30% ethyl acetate in petroleum ether) gave (*R*)-*N*-(*sec*-butyl)-3-(fluoromethyl)-*N*-methyl-4-phenylquinoline-2-carboxamide (7) as a white solid (1.5 g, 95%). NMR spectra showed a 3 : 1 mixture of rotamers. Only signals for the major rotamer are recorded. Mp 146–148 °C; IR (neat) 2972, 1628, 1559, 1485, 1398, 1049, 970 cm^−1^; [*α*]_D_^30^ −12.6 (*c* 0.5, CHCl_3_); ^1^H NMR (400 MHz, CDCl_3_) *δ* 1.05 (3H, t, *J* = 7.4 Hz, CH_2_C*H*_3_), 1.29 (3H, d, *J* = 6.8 Hz, CHC*H*_3_), 1.41–1.78 (2H, m, C*H*_2_CH_3_), 2.77 (3H, s, NC*H*_3_), 4.84–4.95 (1H, m, NC*H*), 5.31 (1H, dd, *J* = 20.8, 10.8 Hz, 3-C*H*HF), 5.44 (1H, dd, *J* 20.8, 10.8 Hz, 3-CH*H*F), 7.31–7.40 (2H, m, 2 × ArH), 7.43–7.58 (5H, m, 5 × ArH), 7.74 (1H, t, *J* = 7.6 Hz, ArH), 8.17 (1H, d, *J* = 8.4 Hz, ArH); ^13^C{^1^H} NMR (101 MHz, CDCl_3_) *δ* 10.9 (CH_3_), 17.3 (CH_3_), 26.5 (CH_3_), 29.9 (CH_2_), 49.9 (CH), 79.2 (CH_2_, ^1^*J*_C–F_ = 162.8 Hz), 123.0 (C, ^2^*J*_C–F_ = 15.1 Hz), 127.0 (CH), 127.1 (C, ^4^*J*_C–F_ = 2.3 Hz), 127.4 (CH, ^5^*J*_C–F_ = 1.2 Hz), 128.5 (2 × CH), 128.7 (CH), 129.6 (2 × CH), 129.7 (CH), 130.4 (CH), 134.8 (C, ^4^*J*_C–F_ = 1.5 Hz), 147.4 (C, ^3^*J*_C–F_ = 2.5 Hz), 150.8 (C, ^3^*J*_C–F_ = 4.7 Hz), 156.6 (C, ^5^*J*_C–F_ = 2.1 Hz), 168.8 (C); MS (ESI) *m*/*z* 373 (M + Na^+^, 100%); HRMS (ESI) *m*/*z*: [M + Na]^+^ calcd for C_22_H_23_FN_2_NaO 373.1687; found 373.1670. Enantiomeric excess was determined by HPLC analysis with a chiralcel AD-H column (hexane : ^*i*^PrOH 97.5 : 2.5, flow rate 1.0 mL min^−1^), *t*_major_ = 30.68 and 32.22 min, *t*_minor_ = 27.15 and 38.38 min; er = 99.5 : 0.5.

#### Ethyl 3-methyl-4-hydroxyquinoline-2-carboxylate (18)^27^

To a solution of aniline (16) (2.25 mL, 24.7 mmol) and diethyl oxalpropionate (17) (4.57 mL, 24.7 mmol) in cyclohexane (200 mL) was added *p*-toluenesulfonic acid (0.03 g, 0.17 mmol) and the mixture heated under reflux at 120 °C using a Dean–Stark condenser for 48 h. The reaction mixture was cooled to room temperature, filtered, washed with hexane (200 mL) and the solvent of the combined filtrate removed under reduced pressure. To this crude oil was added polyphosphoric acid (20.0 g) and the mixture was stirred at 120 °C for 1 h to form a dark brown substance. The substance was cooled to room temperature and 2.4 M aqueous solution of sodium carbonate (100 mL) was added slowly to quench the excess acid and precipitate the product as a yellow solid. Purification by flash column chromatography using a graduated eluent of petroleum ether to petroleum ether/ethyl acetate (1 : 1) afforded ethyl 3-methyl-4-hydroxyquinoline-2-carboxylate (18) as a yellow solid (3.55 g, 60%). Mp 176–178 °C; lit.^27^ 176–178 °C; ^1^H NMR (400 MHz, CDCl_3_) *δ* 1.48 (3H, t, *J* = 7.2 Hz, OCH_2_C*H*_3_), 2.49 (3H, s, 3-CH_3_), 4.52 (2H, q, *J* = 7.2 Hz, OC*H*_2_CH_3_), 7.31–7.37 (2H, m, 2 × ArH), 7.62 (1H, ddd, *J* = 8.4, 5.5, 1.5 Hz, ArH), 8.35 (1H, d, *J* = 8.4 Hz, ArH), 9.08 (1H, s, OH); ^13^C{^1^H} NMR (101 MHz, CDCl_3_) *δ* 11.8 (CH_3_), 14.3 (CH_3_), 63.4 (CH_2_), 117.6 (CH), 122.9 (C), 123.86 (C), 123.92 (CH), 126.7 (CH), 132.8 (CH), 132.9 (C), 138.4 (C), 164.5 (C), 179.9 (C); MS (EI) *m*/*z* 231 (M^+^, 45%), 202 (100), 157 (98), 129 (46), 84 (41); HRMS (EI) *m*/*z*: [M]^+^ calcd for C_13_H_13_NO_3_ 231.0895; found 231.0897.

#### Ethyl 3-methyl-4-bromoquinoline-2-carboxylate (19)^27^

To a solution of ethyl 3-methyl-4-hydroxyquinoline-2-carboxylate (18) (4.55 g, 19.7 mmol) in anhydrous acetonitrile (250 mL) was added phosphorous oxybromide (16.9 g, 59.1 mmol) and anhydrous potassium carbonate (8.16 g, 59.1 mmol). The solution was heated under reflux for 2 h. The reaction mixture was cooled to room temperature and concentrated under reduced pressure to form a brown residue. Water (100 mL) was added and the product was subsequently extracted with ethyl acetate (3 × 100 mL). The organic layer was dried (MgSO_4_) and filtered. The ethyl acetate layer was removed under reduced pressure to form a brown oil from which ethyl 3-methyl-4-bromoquinoline-2-carboxylate (19) precipitated as a brown solid (5.48 g, 95%). Mp 48–50 °C, lit.^27^ 48–50 °C; ^1^H NMR (400 MHz, CDCl_3_) *δ* 1.47 (3H, t, *J* = 7.1 Hz, OCH_2_C*H*_3_), 2.71 (3H, s, 3-CH_3_), 4.54 (2H, q, *J* = 7.1 Hz, OC*H*_2_CH_3_), 7.68 (1H, ddd, *J* = 8.3, 6.9, 1.4 Hz, ArH), 7.75 (1H, ddd, *J* = 8.3, 6.9, 1.4 Hz, ArH), 8.15 (1H, d, *J* = 8.3 Hz, ArH), 8.23 (1H, d, *J* = 8.3 Hz ArH); ^13^C{^1^H} NMR (101 MHz, CDCl_3_) *δ* 14.4 (CH_3_), 20.0 (CH_3_), 62.5 (CH_2_), 127.0 (CH), 128.6 (C), 129.3 (CH), 129.5 (C), 130.0 (CH), 130.3 (CH), 137.4 (C), 146.1 (C), 151.3 (C), 166.6 (C); MS (EI) *m*/*z* 293 (M^+^, 36%), 264 (39), 221 (73), 140 (100), 114 (21), 84 (81); HRMS (EI) *m*/*z*: [M]^+^ calcd for C_13_H_12_^79^BrNO_2_ 293.0051; found 293.0053.

#### Ethyl 3-methyl-4-phenylquinoline-2-carboxylate (20)^27^

To a solution of ethyl 4-bromo-3-methylquinoline-2-carboxylate (19) (3.54 g, 12.0 mmol) in tetrahydrofuran (30 mL) and water (15 mL) was added phenylboronic acid (2.20 g, 18.0 mmol) and potassium phosphate tribasic (5.08 g, 24.0 mmol). The reaction mixture was degassed under argon for 0.2 h. To this solution was added XPhos Pd G2 (0.189 g, 0.240 mmol) and the reaction mixture was stirred at 40 °C for 1.5 h. After cooling to room temperature, the reaction mixture was filtered through a short pad of Celite® and washed with ethyl acetate (100 mL). The filtrate was diluted with water (100 mL) and extracted with ethyl acetate (3 × 100 mL). The combined organic layers were washed with brine (100 mL), dried (MgSO_4_), filtered and concentrated *in vacuo*. Purification by flash column chromatography (20% ethyl acetate in hexane) gave ethyl 3-methyl-4-phenylquinoline-2-carboxylate (20) as a white solid (3.20 g, 91%). Mp 110–112 °C (lit.^27^ 110–112 °C); ^1^H NMR (400 MHz, CDCl_3_) *δ* 1.48 (3H, t, *J* = 7.2 Hz, OCH_2_C*H*_3_), 2.32 (3H, s, 3-CH_3_), 4.55 (2H, q, *J* = 7.2 Hz, OC*H*_2_CH_3_), 7.23–7.27 (2H, m, 2 × ArH), 7.37–7.56 (5H, m, 5 × ArH), 7.67 (1H, ddd, *J* = 8.3, 6.7, 1.4 Hz, ArH), 8.19 (1H, br d, *J* = 8.3 Hz, ArH); ^13^C{^1^H} NMR (101 MHz, CDCl_3_) *δ* 14.4 (CH_3_), 16.9 (CH_3_), 62.2 (CH_2_), 126.2 (CH), 126.3 (C), 127.8 (CH), 128.3 (CH), 128.4 (C), 128.8 (2 × CH), 129.1 (CH), 129.4 (2 × CH), 130.0 (CH), 136.9 (C), 145.8 (C), 148.8 (C), 151.8 (C), 167.6 (C); MS (CI) *m*/*z* 292 (M + H^+^, 100%), 220 (48), 137 (3), 113 (9), 85 (19). HRMS (CI) *m*/*z*: [M + H]^+^ calcd for C_19_H_18_NO_2_ 292.1338; found 292.1336.

#### 3-Methyl-4-phenylquinoline-2-carboxylic acid (21)^24^

To a solution of ethyl 3-methyl-4-phenylquinoline-2-carboxylate (20) (2.87 g, 9.85 mmol) in 50% aqueous ethanol (200 mL) was added sodium hydroxide (1.58 g, 39.4 mmol) and the solution stirred at 80 °C for 1.5 h. The reaction mixture was cooled to room temperature and the ethanol removed *in vacuo* leaving an aqueous solution. This solution was acidified with an aqueous solution of 1 M hydrochloric acid (30 mL) and the product extracted into dichloromethane (100 mL). The organic layer was washed with water (3 × 20 mL), dried (MgSO_4_) and filtered. The organic solvent was removed *in vacuo* to afford 3-methyl-4-phenylquinoline-2-carboxylic acid (21) (2.46 g, 95%), which was used without further purification. Spectroscopic data was consistent with the literature.^24^ Mp 130–132 °C; IR (KBr) 3437, 2931, 1722, 1649, 1590, 1364, 1317, 768 cm^−1^; ^1^H NMR (400 MHz, CDCl_3_) *δ* 2.65 (3H, s, 3-CH_3_), 7.23–7.25 (2H, m, 2 × ArH), 7.43 (1H, d, *J* = 8.3 Hz, ArH), 7.50–7.60 (4H, m, 4 × ArH), 7.75 (1H, ddd, *J* = 8.3, 6.9, 1.2 Hz, ArH), 8.14 (1H, d, *J* = 8.3 Hz, ArH); ^13^C{^1^H} NMR (101 MHz, CDCl_3_) *δ* 17.5 (CH_3_), 126.6 (CH), 128.5 (CH), 128.9 (2 × CH), 129.13 (CH), 129.15 (CH), 129.3 (2 × CH), 129.8 (C), 130.0 (CH), 130.4 (C), 136.5 (C), 143.8 (C), 144.4 (C), 151.5 (C), 164.5 (C); MS (CI) *m*/*z* 264 (M + H^+^, 100%), 220 (19), 188 (3), 85 (27); HRMS (CI) *m*/*z*: [M + H]^+^ calcd for C_17_H_14_NO_2_ 264.1025; found 264.1027.

#### (*R*)-(*N-sec*-Butyl)-3-methyl-4-phenylquinoline-2-carboxamide (22)^36^

To a solution of 3-methyl-4-phenylquinoline-2-carboxylic acid (21) (2.54 g, 9.65 mmol) in anhydrous *N*,*N*-dimethylformamide (250 mL) was added *O*-(benzotriazol-1-yl)-*N*,*N*,*N*′,*N*′-tetramethyluronium hexafluorophosphate (5.49 g, 14.5 mmol) and *N*,*N*′-diisopropylethylamine (3.40 mL, 19.3 mmol) under argon. The reaction mixture was stirred at room temperature for 0.5 h before the addition of (*R*)-(−)-*sec*-butylamine (1.10 mL, 10.6 mmol) and then heated to 40 °C for 4 h. The reaction mixture was cooled to room temperature and diluted with ethyl acetate (300 mL) and washed with water (3 × 200 mL) and brine (200 mL). The organic layer was dried (MgSO_4_), filtered and concentrated *in vacuo* to give a brown oil. Purification by flash column chromatography (20% ethyl acetate in petroleum ether) gave (*R*)-(*N-sec*-butyl)-3-methyl-4-phenylquinoline-2-carboxamide (22) as a white solid (2.81 g, 91%). Mp 152–154 °C, lit.^36^ 157–158 °C; IR (KBr) 3282, 2967, 1639, 1535, 1443, 1156, 761 cm^−1^; [*α*]_D_^25^ −26.7 (*c* 1.0, CHCl_3_); ^1^H NMR (400 MHz, CDCl_3_) *δ* 1.03 (3H, t, *J* = 7.4 Hz, CHCH_2_C*H*_3_), 1.33 (3H, d, *J* = 6.6 Hz, CHC*H*_3_), 1.61–1.75 (2H, m, C*H*_2_CH_3_), 2.56 (3H, s, 3-CH_3_), 4.08–4.20 (1H, m, C*H*CH_3_), 7.21–7.25 (2H, m, 2 × ArH), 7.35 (1H, d, *J* = 8.3 Hz, ArH), 7.40–7.55 (3H, m, 2 × ArH), 7.65 (1H, ddd, *J* = 8.3, 6.8, 1.4 Hz, ArH), 7.90 (1H, d, *J* = 8.3 Hz, ArH), 8.09 (1H, d, *J* = 8.3 Hz, ArH); ^13^C{^1^H} NMR (101 MHz, CDCl_3_) *δ* 10.6 (CH_3_), 17.6 (CH_3_), 20.5 (CH_3_), 29.9 (CH_2_), 46.8 (CH), 126.2 (CH), 127.5 (CH), 127.9 (CH), 128.6 (C), 128.6 (2 × CH), 128.7 (CH and C), 129.3 (2 × CH), 129.4 (CH), 137.3 (C), 144.7 (C), 149.5 (C), 150.1 (C), 166.2 (C); MS (CI) *m*/*z* 319 (M + H^+^, 100%), 220 (19), 202 (5), 148 (6), 113 (16), 85 (77); HRMS (CI) *m*/*z*: [M + H]^+^ calcd for C_21_H_23_N_2_O 319.1810; found 319.1809.

#### (*S*)-(*N-sec*-Butyl)-3-methyl-4-phenylquinoline-2-carboxamide (23)

The reaction was performed as described for the (*R*)-enantiomer above using 3-methyl-4-phenylquinoline-2-carboxylic acid (21) (0.527 g, 2.00 mmol), *O*-(benzotriazol-1-yl)-*N*,*N*,*N*′,*N*′-tetramethyluronium hexafluorophosphate (1.14 g, 3.00 mmol), *N*,*N*-diisopropylethylamine (0.699 mL, 4.00 mmol) and (*S*)-(+)-*sec*-butylamine (0.223 mL, 2.20 mmol) in *N*,*N*-dimethylformamide (20 mL). Purification by flash column chromatography (20% ethyl acetate in hexane) gave (*S*)-(*N-sec*-butyl)-3-methyl-4-phenylquinoline-2-carboxamide (23) as a white solid (0.602 g, 93%). [*α*]_D_^16^ +23.5 (*c* 0.1, CHCl_3_); all other data as described for the (*R*)-enantiomer above.

#### (*R*)-(*N-sec*-Butyl)-*N*-methyl-3-methyl-4-phenylquinoline-2-carboxamide (24)^36^

To a solution of (*R*)-(*N-sec*-butyl)-3-methyl-4-phenylquinoline-2-carboxamide (22) (2.81 g, 8.82 mmol) in tetrahydrofuran (176 mL) was added sodium hydride (60% dispersion in mineral oil, 0.710 g, 17.6 mmol). The mixture was stirred at room temperature for 0.5 h, before the addition of iodomethane (2.75 mL, 44.1 mmol). The resultant solution was stirred at room temperature for 3 h, before being quenched by the addition of water (100 mL). The aqueous phase was extracted with diethyl ether (3 × 100 mL). The combined organic layers were washed with a 10% aqueous solution of sodium thiosulfate (10 mL), brine (10 mL), dried (Na_2_SO_4_), filtered and concentrated *in vacuo*. Purification by flash column chromatography (20–50% ethyl acetate in hexane) gave (*R*)-(*N-sec*-butyl)-*N*-methyl-3-methyl-4-phenylquinoline-2-carboxamide (24) as a white solid (2.75 g, 94%). NMR spectroscopy showed a 1.4 : 1 mixture of rotamers. Only signals for the major rotamer are recorded. Mp 114–117 °C, lit.^36^ 117–118 °C; [α]_D_^23^ −6.3 (*c* 1.0, CHCl_3_); ^1^H NMR (400 MHz, CDCl_3_) *δ* 0.86 (3H, t, *J* = 7.3 Hz, CH_2_C*H*_3_), 1.24 (3H, d, *J* = 6.6 Hz, CHC*H*_3_), 1.55–1.70 (2H, m, C*H*_2_CH_3_), 2.23 (3H, s, 3-CH_3_), 3.05 (3H, s, NCH_3_), 3.42–3.53 (1H, m, C*H*CH_3_), 7.25–7.31 (2H, m, 2 × ArH), 7.38–7.44 (2H, m, 2 × ArH), 7.47–7.57 (3H, m, 3 × ArH), 7.62–7.67 (1H, m, ArH), 8.10 (1H, d, *J* = 8.3 Hz, ArH); ^13^C{^1^H} NMR (101 MHz, CDCl_3_) *δ* 11.3 (CH_3_), 16.4 (CH_3_), 18.6 (CH_3_), 25.5 (CH_3_), 27.2 (CH_2_) 49.6 (CH) 125.3 (C), 125.9 (CH), 126.8 (2 × CH), 127.4 (C), 128.0 (CH), 128.6 (CH), 128.7 (CH), 129.2 (2 × CH), 129.4 (CH), 136.8 (C), 145.8 (C), 148.0 (C), 156.1 (C), 169.7 (C); MS (EI) *m*/*z* 332 (M^+^, 32%), 303 (35), 275 (22), 218 (89), 189 (25), 86 (100); HRMS (EI) *m*/*z*: [M]^+^ calcd for C_22_H_24_N_2_O 332.1889; found 332.1888.

#### (*S*)-(*N-sec*-Butyl)-*N*-methyl-3-methyl-4-phenylquinoline-2-carboxamide (25)

The reaction was performed as described for the (*R*)-enantiomer above using (*S*)-(*N-sec*-butyl)-3-methyl-4-phenylquinoline-2-carboxamide (23) (0.726 g, 2.28 mmol), sodium hydride (60% dispersion in mineral oil) (0.182 g, 4.56 mmol) and methyl iodide (0.710 mL, 11.4 mmol) in tetrahydrofuran (45 mL). Purification by flash column chromatography (20–50% ethyl acetate in hexane) gave (*S*)-(*N-sec*-butyl)-*N*-methyl-3-methyl-4-phenylquinoline-2-carboxamide (25) as a white solid (0.664 g, 88%). [α]_D_^17^ +9.3 (*c* 0.1, CHCl_3_); all other data as described for the (*R*)-enantiomer above.

#### (*R*)-3-Bromomethyl-(*N-sec*-butyl)-*N*-methyl-4-phenylquinoline-2-carboxamide (26)

To a stirred, degassed solution of (*R*)-(*N-sec*-butyl)-*N*-methyl-3-methyl-4-phenylquinoline-2-carboxamide (24) (2.70 g, 8.12 mmol) in chloroform (300 mL) under argon was added *N*-bromosuccinimide (2.17 g, 12.2 mmol) and dibenzoyl peroxide (0.20 g, 0.812 mmol) and the solution heated under reflux for 6 h. A further portion of *N*-bromosuccinimide (1.00 g, 5.61 mmol) was then added and the solution heated under reflux for a further 18 h. The reaction mixture was cooled to room temperature, filtered and the solvent removed *in vacuo*. The crude residue was then diluted with ethyl acetate (100 mL) and washed with water (3 × 100 mL). The organic layer was dried (MgSO_4_), filtered and concentrated *in vacuo*. Purification by flash column chromatography (0–5% ethyl acetate in dichloromethane) afforded (*R*)-3-bromomethyl-(*N-sec*-butyl)-*N*-methyl-4-phenylquinoline-2-carboxamide (26) as an orange solid (2.83 g, 85%). NMR spectra showed a 2 : 1 mixture of rotamers. Only signals for the major rotamer are recorded. Mp 160–164 °C; IR (KBr) 2966, 1630, 1483, 1396, 1045, 760 cm^−1^; [*α*]_D_^28^ −9.0 (*c* 1.0, CHCl_3_); ^1^H NMR (400 MHz, CDCl_3_) *δ* 1.09 (3H, t, *J* = 7.4 Hz, CH_2_C*H*_3_), 1.33 (3H, d, *J* = 6.8 Hz, CHC*H*_3_), 1.52–1.67 (2H, m, C*H*_2_CH_3_), 2.86 (3H, s, NCH_3_), 4.61 (1H, d, *J* = 10.2 Hz, 3-C*H*HBr), 4.68 (1H, d, *J* = 10.2 Hz, 3-CH*H*Br), 4.83 (1H, sextet, *J* = 6.8 Hz, C*H*CH_3_), 7.38–7.48 (4H, m, 4 × ArH), 7.52–7.60 (3H, m, 3 × ArH), 7.72 (1H, ddd, *J* = 8.3, 6.7, 1.5 Hz, ArH), 8.07–8.14 (1H, m, ArH); ^13^C{^1^H} NMR (101 MHz, CDCl_3_) *δ* 11.3 (CH_3_), 17.2 (CH_3_), 26.7 (CH_2_), 27.9 (CH_2_), 30.6 (CH_3_), 50.3 (CH), 126.4 (C), 126.9 (2 × CH), 127.6 (CH), 128.7 (2 × CH), 128.8 (CH), 129.2 (CH), 129.3 (CH), 129.7 (CH), 135.1 (C), 135.12 (C), 146.6 (C), 149.5 (C), 156.1 (C), 168.6 (C); MS (EI) *m*/*z* 410 (M^+^, 46%), 330 (27), 217 (57), 189 (28), 86 (100); HRMS (EI) *m*/*z*: [M]^+^ calcd for C_22_H_23_^79^BrN_2_O 410.0994; found 410.0995.

#### (*S*)-3-Bromomethyl-(*N-sec*-butyl)-*N*-methyl-4-phenylquinoline-2-carboxamide (27)

The reaction was performed as described for the (*R*)-enantiomer above using (*S*)-(*N-sec*-butyl)-*N*-methyl-3-methyl-4-phenylquinoline-2-carboxamide (25) (0.440 g, 1.32 mmol), *N*-bromosuccinimide (0.353 g, 1.99 mmol) and dibenzoyl peroxide (0.0426 g, 0.132 mmol) in chloroform (10 mL). Purification by flash column chromatography (2.5–5% ethyl acetate in dichloromethane) gave (*S*)-3-bromomethyl-(*N-sec*-butyl)-*N*-methyl-4-phenylquinoline-2-carboxamide (27) as a white solid (0.426 g, 78%). [*α*]_D_^17^ +11.4 (*c* 0.1, CHCl_3_); all other data as described for the (*R*)-enantiomer above.

#### (*R*)-(*N-sec*-Butyl)-3-fluoromethyl-*N*-methyl-4-phenylquinoline-2-carboxamide (LW223, 7)

To a solution of 18-crown-6 (1.6 g, 6.0 mmol) in anhydrous acetonitrile (100 mL) under argon was added potassium fluoride (1.8 g, 31 mmol) and the resulting suspension stirred at room temperature for 0.5 h. A solution of (*R*)-3-bromomethyl-(*N-sec*-butyl)-*N*-methyl-4-phenylquinoline-2-carboxamide (26) (2.5 g, 6.0 mmol) in acetonitrile : dichloromethane (2 : 1, 300 mL) was then added dropwise and the reaction mixture heated under reflux for 72 h. Upon completion, the reaction mixture was cooled to ambient temperature and water (100 mL) was added. The solution was extracted with dichloromethane (3 × 200 mL), dried (MgSO_4_), filtered and concentrated *in vacuo*. Purification by flash column chromatography (30% ethyl acetate in hexane) gave (*R*)-*N*-(*sec*-butyl)-3-(fluoromethyl)-*N*-methyl-4-phenylquinoline-2-carboxamide (7) as a white solid (1.6 g, 73%). Data as described above.

#### (*S*)-(*N-sec*-Butyl)-3-(fluoromethyl)-*N*-methyl-4-phenylquinoline-2-carboxamide (*S*-LW223, 8)

The reaction was performed as described for the (*R*)-enantiomer above using (*S*)-(*N-sec*-butyl)-3-bromomethyl-*N*-methyl-4-phenylquinoline-2-carboxamide (27) (0.259 g, 0.630 mmol), 18-crown-6 (0.188 g, 0.713 mmol) and potassium fluoride (0.207 g, 3.57 mmol) in acetonitrile (15 mL). The mixture was stirred under reflux for 24 h. Further portions of 18-crown-6 (0.188 g, 0.713 mmol) and potassium fluoride (0.138 g, 2.38 mmol) were then added and the reaction mixture heated under reflux for a further 24 h. Purification by flash column chromatography (30% ethyl acetate in hexane) gave (*S*)-(*N-sec*-butyl)-3-(fluoromethyl)-*N*-methyl-4-phenylquinoline-2-carboxamide (8) as a white solid (0.176 g, 80%). [*α*]_D_^17^ +14.1 (*c* 0.1, CHCl_3_); all other data as described for the (*R*)-enantiomer above.

#### (*R*)-(*N-sec*-Butyl)-3-chloromethyl-*N*-methyl-4-phenylquinoline-2-carboxamide (28)

To a solution of (*R*)-3-bromomethyl-(*N-sec*-butyl)-*N*-methyl-4-phenylquinoline-2-carboxamide (26) (0.500 g, 1.22 mmol) in dry tetrahydrofuran (10 mL) was added lithium chloride (0.160 g, 3.66 mmol) and the reaction mixture stirred at room temperature for 16 h. The reaction was quenched with water (30 mL) and extracted into ethyl acetate (3 × 30 mL). The organic layers were combined and washed with brine (90 ml), dried (MgSO_4_), filtered and concentrated *in vacuo*. The product was purified by flash column chromatography (dichloromethane/ethyl acetate, 95 : 5) to afford (*R*)-3-chloromethyl-(*N-sec*-butyl)-*N*-methyl-4-phenylquinoline-2-carboxamide (28) as a white solid (0.331 g, 74%). NMR spectra showed a 1.5 : 1 mixture of rotamers. Only signals for the major rotamer are recorded. Mp 140–142 °C; IR (neat) 2970, 1620, 1481, 1404, 1219, 748 cm^−1^; [*α*]_D_^24^ −11.6 (*c* 1.0, CHCl_3_); ^1^H NMR (400 MHz, CDCl_3_) *δ* 1.08 (3H, t, *J* = 7.4 Hz, CH_2_C*H*_3_), 1.30 (3H, d, *J* = 6.8 Hz, CHC*H*_3_), 1.49–1.79 (2H, m, C*H*_2_CH_3_), 2.84 (3H, s, NCH_3_), 4.67 (1H, d, *J* = 10.6 Hz, 3-C*H*H), 4.72 (1H, d, *J* = 10.6 Hz, 3-CH*H*), 4.82–4.92 (1H, m, C*H*CH_3_), 7.36–7.61 (7H, m, ArH), 7.69–7.75 (1H, m, ArH), 8.11 (1H, dd, *J* 9.0, 8.4 Hz, ArH); ^13^C NMR (101 MHz, CDCl_3_) *δ* 11.1 (CH_3_), 17.1 (CH_3_), 26.6 (CH_2_), 30.4 (CH_3_), 40.4 (CH_2_), 50.1 (CH), 125.8 (C), 126.8 (CH), 127.2 (C), 127.4 (CH), 128.5 (2 × CH), 128.7 (CH), 129.4 (CH), 129.6 (2 × CH), 130.1 (CH), 134.9 (C), 146.6 (C), 149.5 (C), 156.1 (C), 168.5 (C); MS (ESI) *m*/*z* 389 (M + Na^+^, 100%); HRMS (ESI) *m*/*z*: [M + H]^+^ calcd for C_22_H_23_^35^ClN_2_NaO 389.1391; found 389.1381.

#### *In vitro* human competition binding assays

All studies using human tissue were conducted and approved in accordance with the National Health Service Research Ethics Committee Southeast Scotland (NHS-RECSE, Edinburgh Brain and Tissue Bank, 16/ES/0084). Informed consents were obtained from human participants of this study. The Edinburgh Brain and Tissue code and BNN reference numbers for all new tissue samples used in this study are: SD022/13 (BBN_15222), SD038/13 (BBN_18391), SD037/14 (BBN_23395), SD051/14 (BBN_24340), SD012/15 (BBN_25055), SD032/16 (0.01.29084), SD027/12 (BBN_13410), SD038/12 (BBN_4176), SD006/14 (BBN_20593), SD008/14 (BBN_20592), SD020/14 (BBN_22611), SD011/15 (BBN_24781), SD023/12 (BBN_3785), SD029/13 (BBN_15809), SD036/13 (BBN_18392), SD046/13 (BBN_18407), SD034/14 (BBN_22628) and SD031/15 (001.26124). Briefly, 250 μg protein per mL in buffer (200 μL) was added to 1 nM [^3^H]PK11195 (100 μL) (PerkinElmer, USA), together with (*S*)-LW223 (100 μL) at 14 different concentrations (0.001–3000 nM) for 1.5 h at 4 °C. The data on (*R*)-LW223 and PK11195 (Sigma-Aldrich, USA) binding was generated as part of our previous study.^17^ Binding was terminated with the addition of ice cold buffer before being immediately filtered over Whatman GF/B filter paper (Whatman, UK) pre-treated with 0.3% polyethylenimine (Sigma-Aldrich, USA) using a Brandel harvester (Brandel, USA). Filter paper was then removed, placed into Optiphase HiSafe 3 (2.5 mL) (Perkin Elmer, USA) and counted 48 h later on a Hidex 300 SL (Hidex, Finland). Saturation assays were previously performed to determine the *K*_d_ of PK11195 as 14.0 nM.^17^ All binding assays were performed in triplicate. GraphPad Prism version 9 (GraphPad Software, USA) was used to fit all binding affinity curves. Curves were fitted using a one-site binding model. Normalised mean% SB of each group (HAB, MAB or LAB) and a *K*_d_ value of 14.0 nM was used to calculate LAB : HAB ratio. Affinity values (*K*_i_) were calculated by fitting individual tissue samples again with a *K*_d_ value of 14.0 nM.

#### Docking studies

All computational experiments were conducted on a Dell Latitude 5420 11th Gen Intel(R) Core(TM) i7-1185G7. The X-ray structure of PK11195-bound TSPO from *Bacillus cereus* was obtained from the Protein Database (PDB code: 4RYI, http://www.rcsb.org).^32^ (*R*)-LW223 and (*S*)-LW223 were drawn using ChemDraw 22.2.0. The three-dimensional, energetically minimised conformer of each compound was then obtained using Mercury software from the Cambridge Crystallographic Data Centre (CCDC). Using Hermes GOLD software from the CCDC, a docking was configured with the *Bc*TSPO/PK11195 complex (PDB code: 4RYI) in which the bound PK11195 ligand was extracted, and hydrogen atoms were added to the protein. No water molecules were present within the X-ray structure of the protein, and no further changes were made. The binding site was defined using the previously extracted PK11195 ligand with a radius of 10.0 Å. The energetically minimised conformer of (*R*)- and (*S*)-LW223 were specified as the ligands for docking into *Bc*TSPO and the default scoring function of the GOLD software, ChemPLP, was used to score the docking solutions. Additional amendments to default settings were made such as no early termination and search efficiency was specified as >200%. The docking solutions were then analysed within Hermes and UCSF ChimeraX 1.8 software.

## Conflicts of interest

There are no conflicts to declare.

## Supplementary Material

MD-OLF-D5MD00930H-s001

## Data Availability

The data supporting this article have been included as part of the supplementary information (SI). This includes the chiral HPLC trace for (*R*)-LW223 and ^1^H and ^13^C NMR spectra of all compounds. Supplementary information: chiral HPLC trace for LW223, other views of docked (*S*)- and (*R*)-LW223, physicochemical procedure and full data, ^1^H and ^13^C NMR spectra of all compounds. See DOI: https://doi.org/10.1039/d5md00930h.
