# Supplementary material for: Synthesis and evaluation of enantiomeric quinoline-2-carboxamides: positron emission tomography imaging agents for the translocator protein
Source: RSC Med Chem. 2025 Dec 11;17(2):987–99. doi: 10.1039/d5md00930h (PMC12767876; doi:10.1039/d5md00930h)

Supplementary Information (SI) for RSC Medicinal Chemistry.
This journal is © The Royal Society of Chemistry 2025

Supporting Information for:

Synthesis and Evaluation of Enantiomeric Quinoline-2-carboxamides: Positron Emission Tomography Imaging Agents for the Translocator Protein

Lachlan J. N. Waddell,[a] Mark G. MacAskill,[b,c] Holly McErlain,[a] Timaeus E. F. Morgan,[a] Lewis Williams,[a] Victoria J. M. Reid,[b,c] Anna Beyger,[a] Sally L. Pimlott,[d] Adriana A. S. Tavares[b,c] and Andrew Sutherland[a]*

[a]School of Chemistry, University of Glasgow, University Avenue, Glasgow, G12 8QQ, U.K.
[b]Edinburgh Imaging, University of Edinburgh, 47 Little France Crescent, Edinburgh, EH16 4TJ, U.K.
[c]University/BHF Centre for Cardiovascular Sciences, University of Edinburgh, 47 Little France Crescent, Edinburgh, EH16 4TJ, U.K. [d]West of Scotland PET Centre, Greater Glasgow and Clyde NHS Trust, Glasgow, G12 OYN, U.K.

Table of Contents

1. Chiral HPLC Trace for (*R*)-LW223 S2
2. Other Docked Views of (*R*)- and (*S*)-LW223 S3–S4
3. Procedure and Replicates for Physicochemical Data S5
4. ^{1}H and ^{13}C NMR Spectra for all Compounds S6–S33

1. Chiral HPLC Trace for (*R*)-LW223.

The chiral HPLC method was calibrated with the corresponding racemic mixture and performed using an AD-H column, 2.5% isopropanol in hexane and a 1 mL min^{-1} flow rate. (Note: each enantiomer of LW223 appears as a mixture of amide rotamers by HPLC).

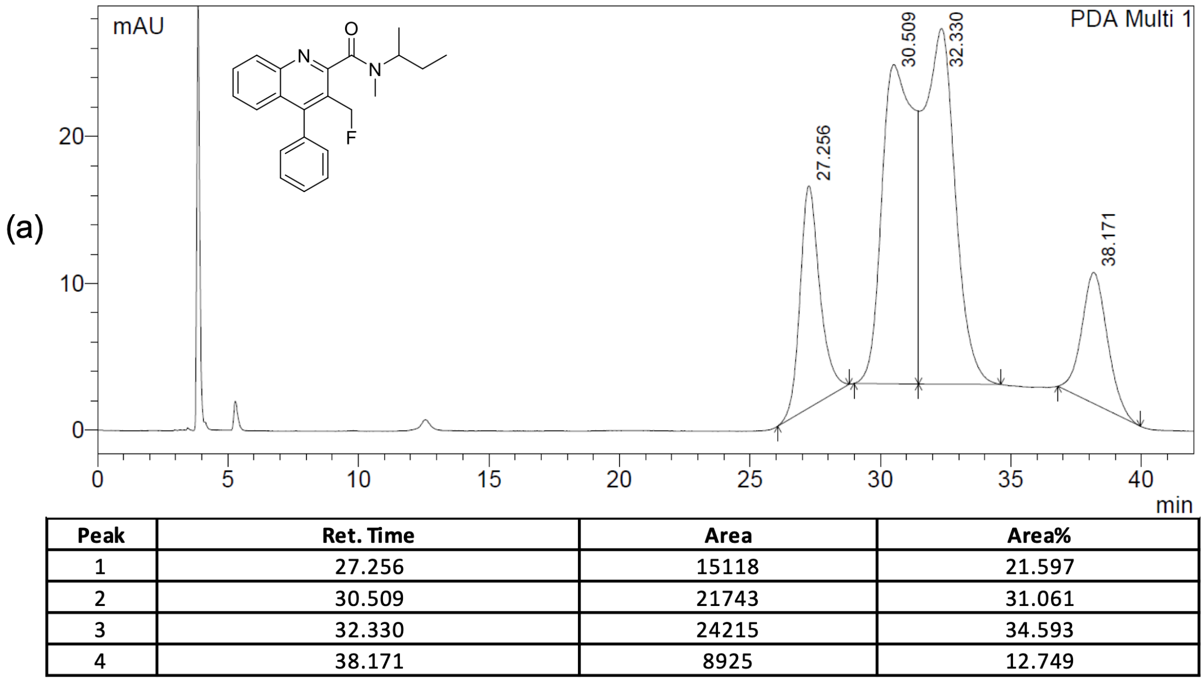

Peak	Ret. Time	Area	Area%
1	27.256	15118	21.597
2	30.509	21743	31.061
3	32.330	24215	34.593
4	38.171	8925	12.749

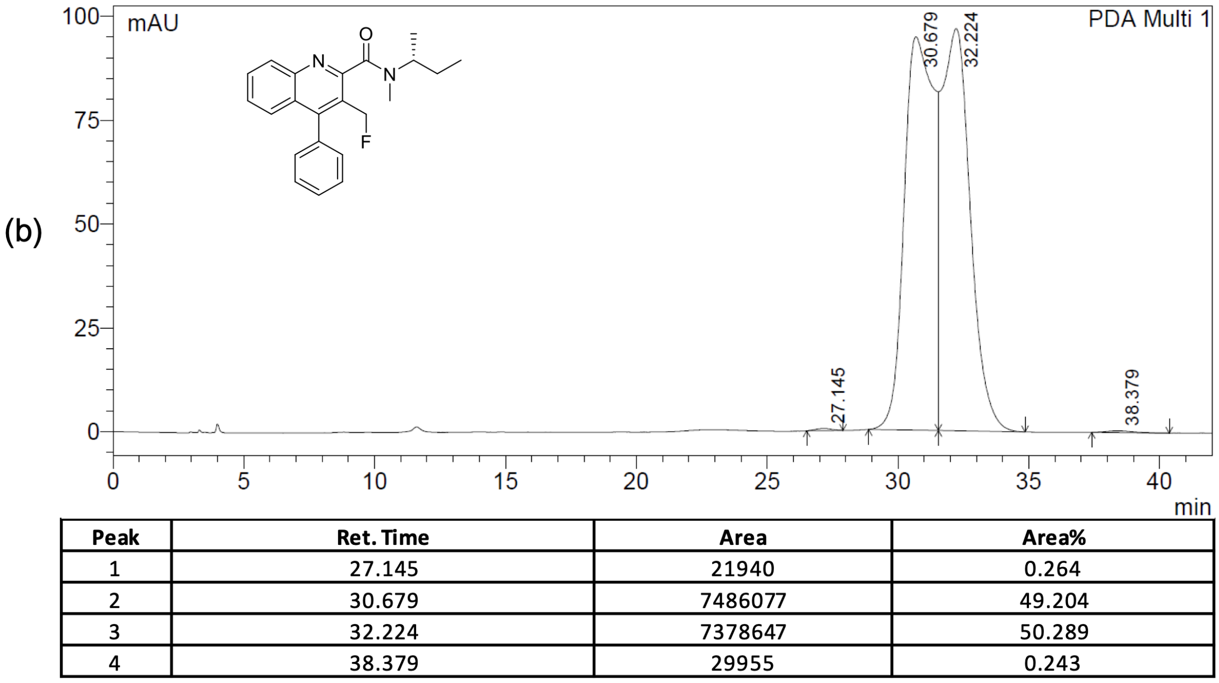

Peak	Ret. Time	Area	Area%
1	27.145	21940	0.264
2	30.679	7486077	49.204
3	32.224	7378647	50.289
4	38.379	29955	0.243

Figure S1: Chiral HPLC of (a) racemic LW223 and (b) (*R*)-LW223 showing a 99.5:0.5 er.

2. Other Docked Views of (*R*)- and (*S*)-LW223.

(*R*)-LW223:

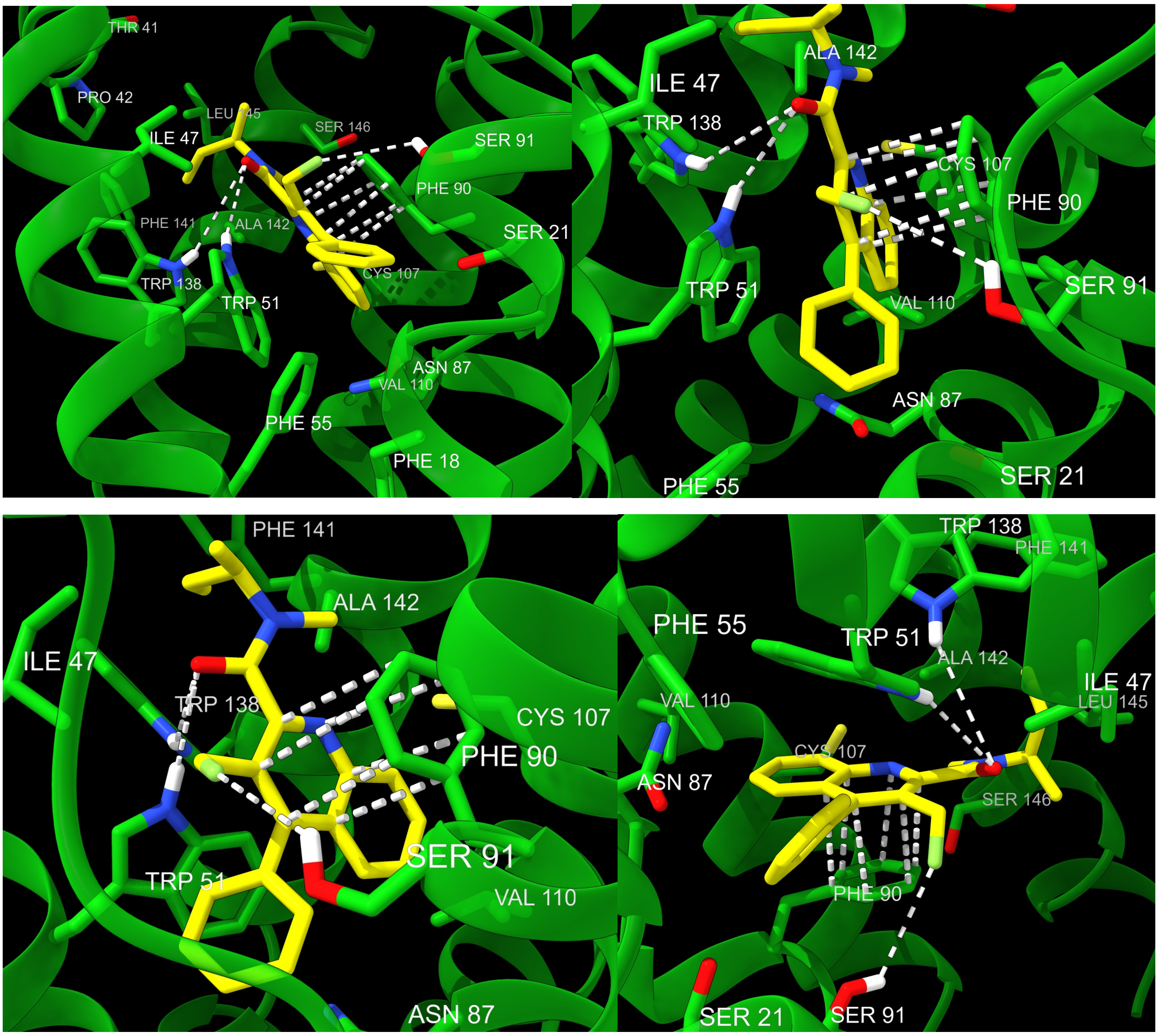

(*S*)-LW223:

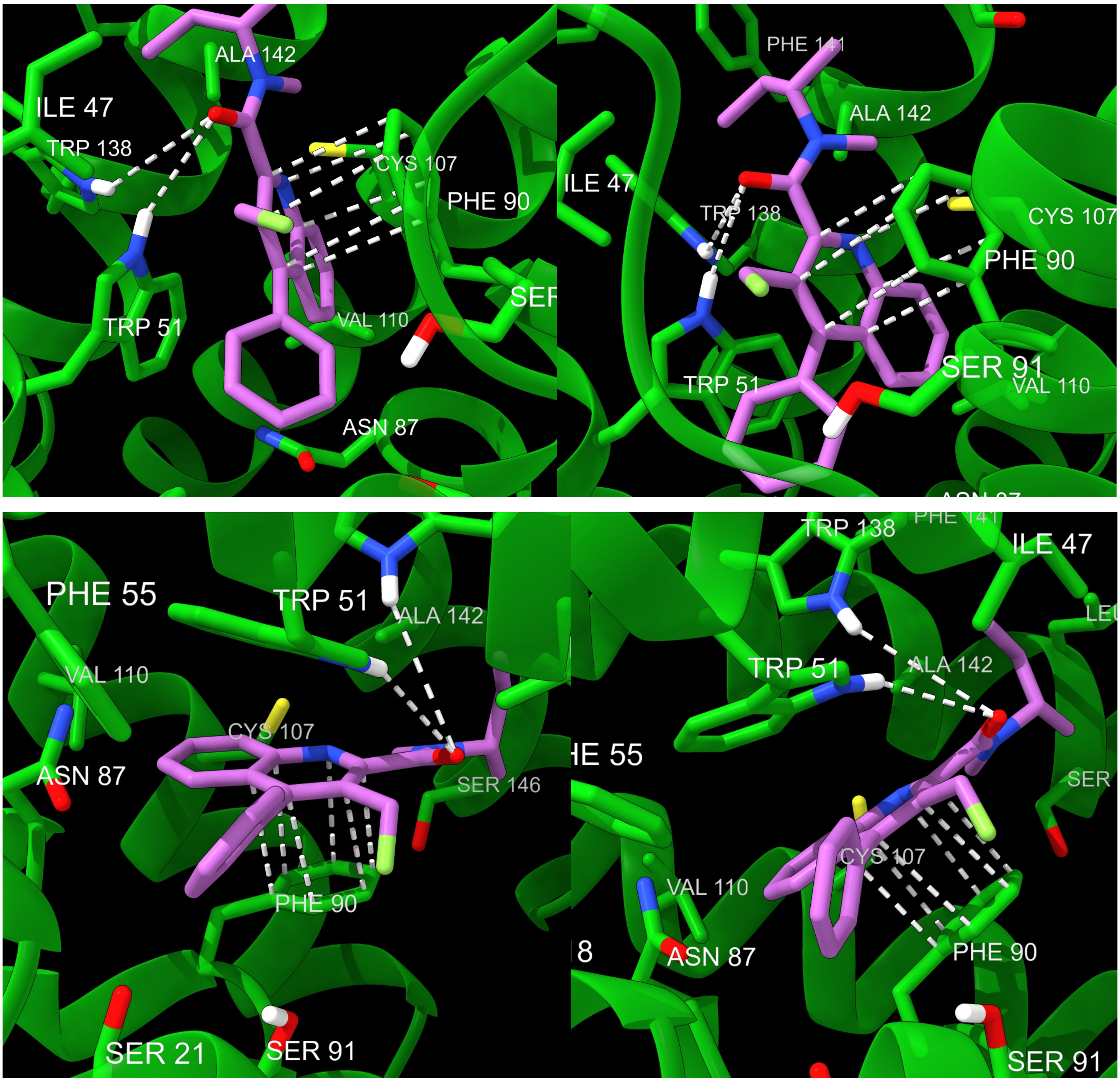
ALA 142
ILE 47
TRP 138
CYS 107
PHE 90
TRP 51
VAL 110
ASN 87
PHE 141
ALA 142
ILE 47
TRP 138
CYS 107
PHE 90
TRP 51
SER 91
VAL 110
PHE 55
TRP 51
ALA 142
VAL 110
CYS 107
ASN 87
SER 146
PHE 90
SER 21
SER 91
TRP 138
PHE 141
ILE 47
TRP 51
ALA 142
CYS 107
VAL 110
ASN 87
PHE 90
SER 91

3. Procedure and Replicates for Physicochemical Data

All physicochemical analyses were performed using a Dionex Ultimate 3000 series, and data acquisition and processing performed using Chromeleon 6.8 Chromatography software. Standard and test compounds were dissolved in 1:1 organic/aqueous phases and prepared to a concentration of 0.5 mg/mL. The HPLC system was set to 25 °C, and UV detection achieved using a diode array detector (190–800 nm). Analysis was performed using 5 μL sample injections.

	**Log *P*[a]**		**P_m[b]**			**K_m[b]**			**%PPM[c]**		
	**1**	**2**	**1**	**2**	**3**	**1**	**2**	**3**	**1**	**2**	**3**
**PK11195**	3.85	4.11	0.54	0.50	0.52	189	186	178	90	94	91
**AB5186**	3.74	3.73	0.36	0.34	0.35	129	122	120	90	88	91
**LW223**	4.17	4.09	0.57	0.56	0.54	202	195	188	91	95	89

[a] Determined using C_{18} column. [b] Determined using immobilised artificial membrane (IAM) column. [c] Determined using human serum albumin (HAS) coated column.

4. ^{1}H and ^{13}C NMR Spectra for all Compounds

^{1}H NMR (400 MHz, $CDCl_3$)

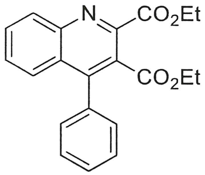

10

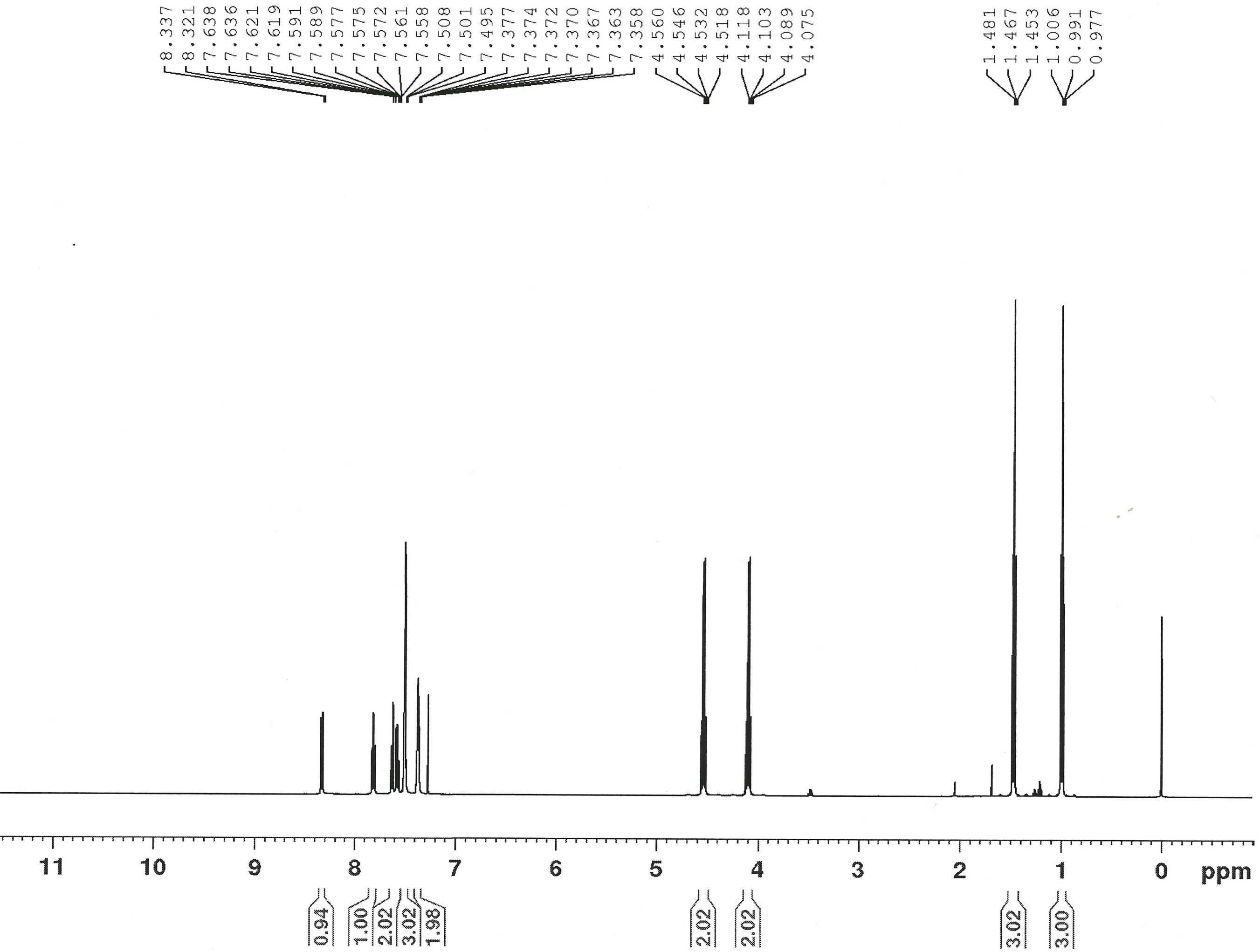

$^{13}C\{^{1}H\}$ NMR (101 MHz, $CDCl_3$)

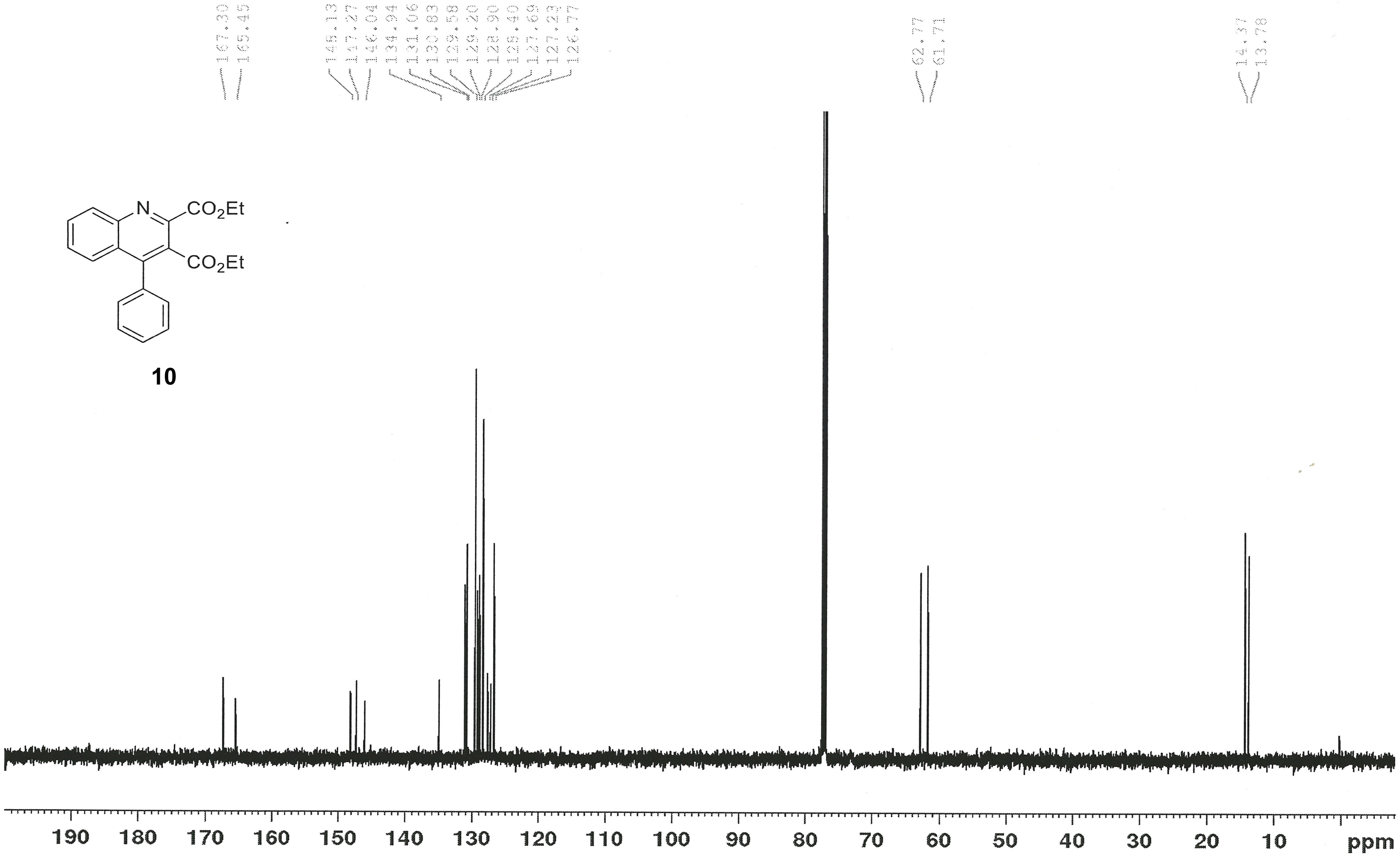

^{1}H NMR (400 MHz, $CDCl_3$)

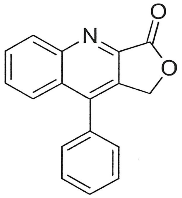

12

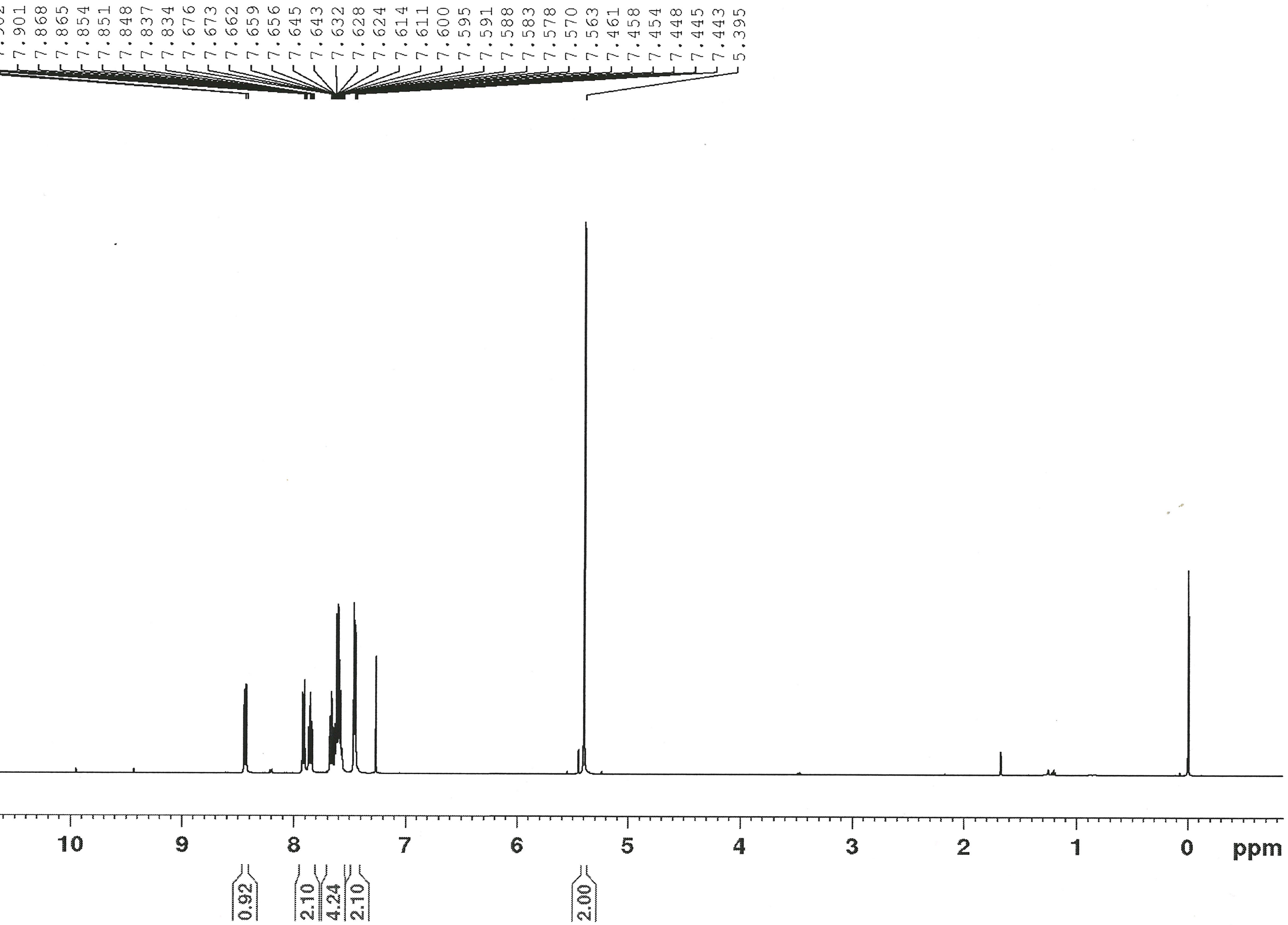

$^{13}C\{^{1}H\}$ NMR (101 MHz, $CDCl_3$)

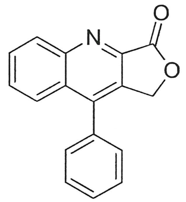

12

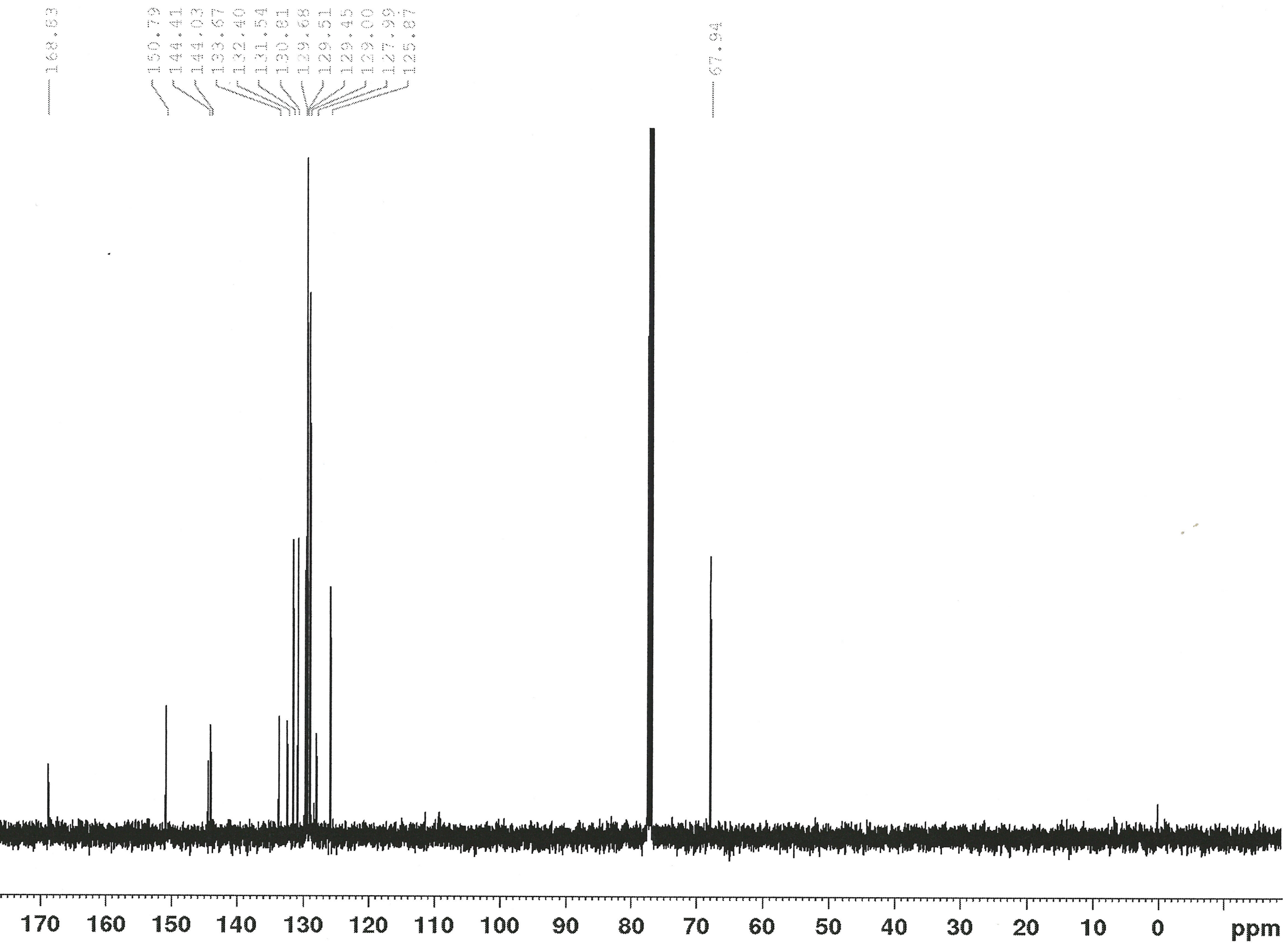

^{1}H NMR (400 MHz, CDCl$_3$)

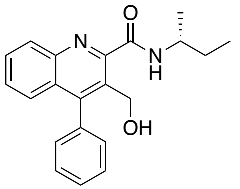

13

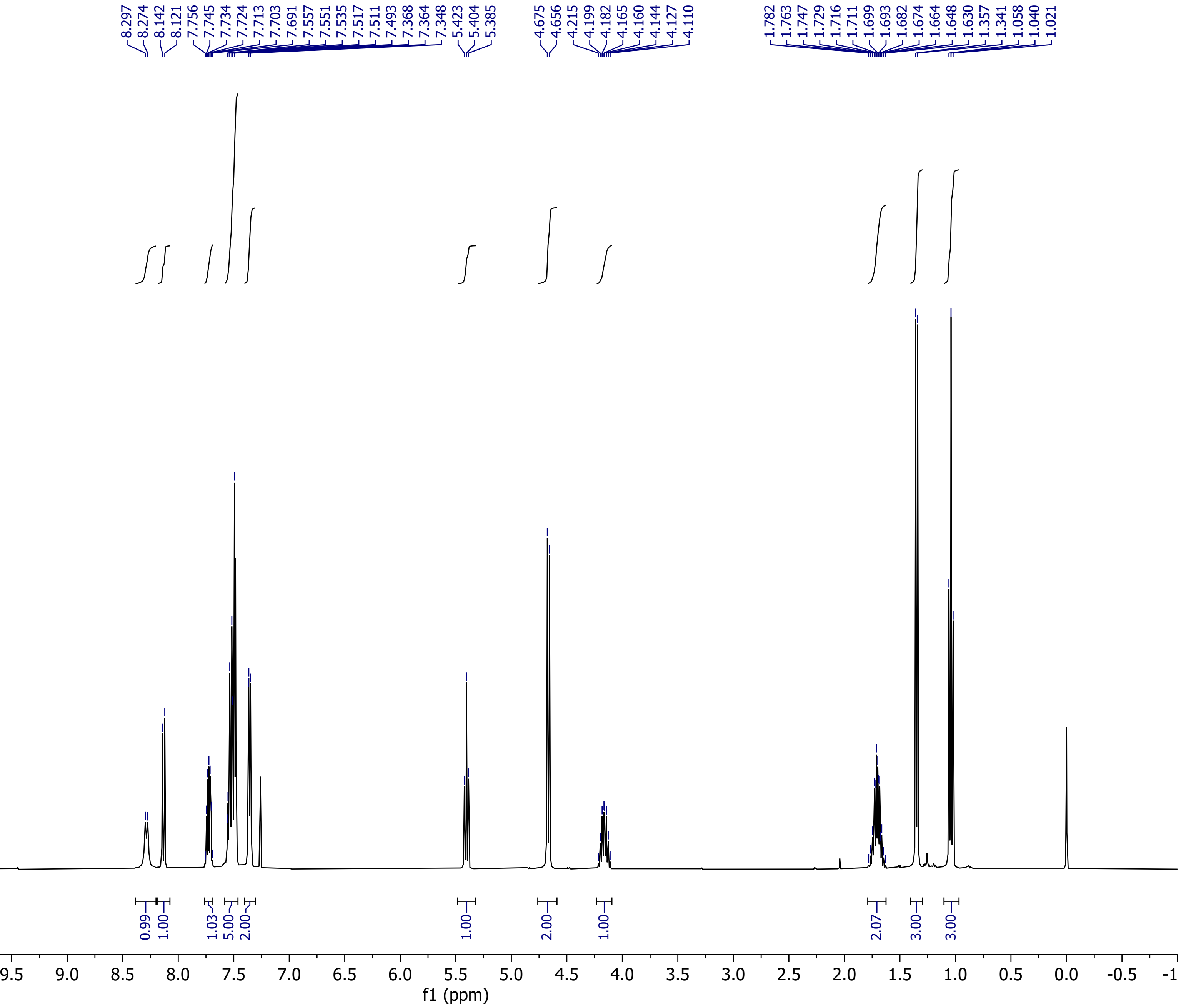

$^{13}C\{^{1}H\}$ NMR (101 MHz, $CDCl_3$)

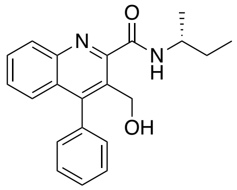

13

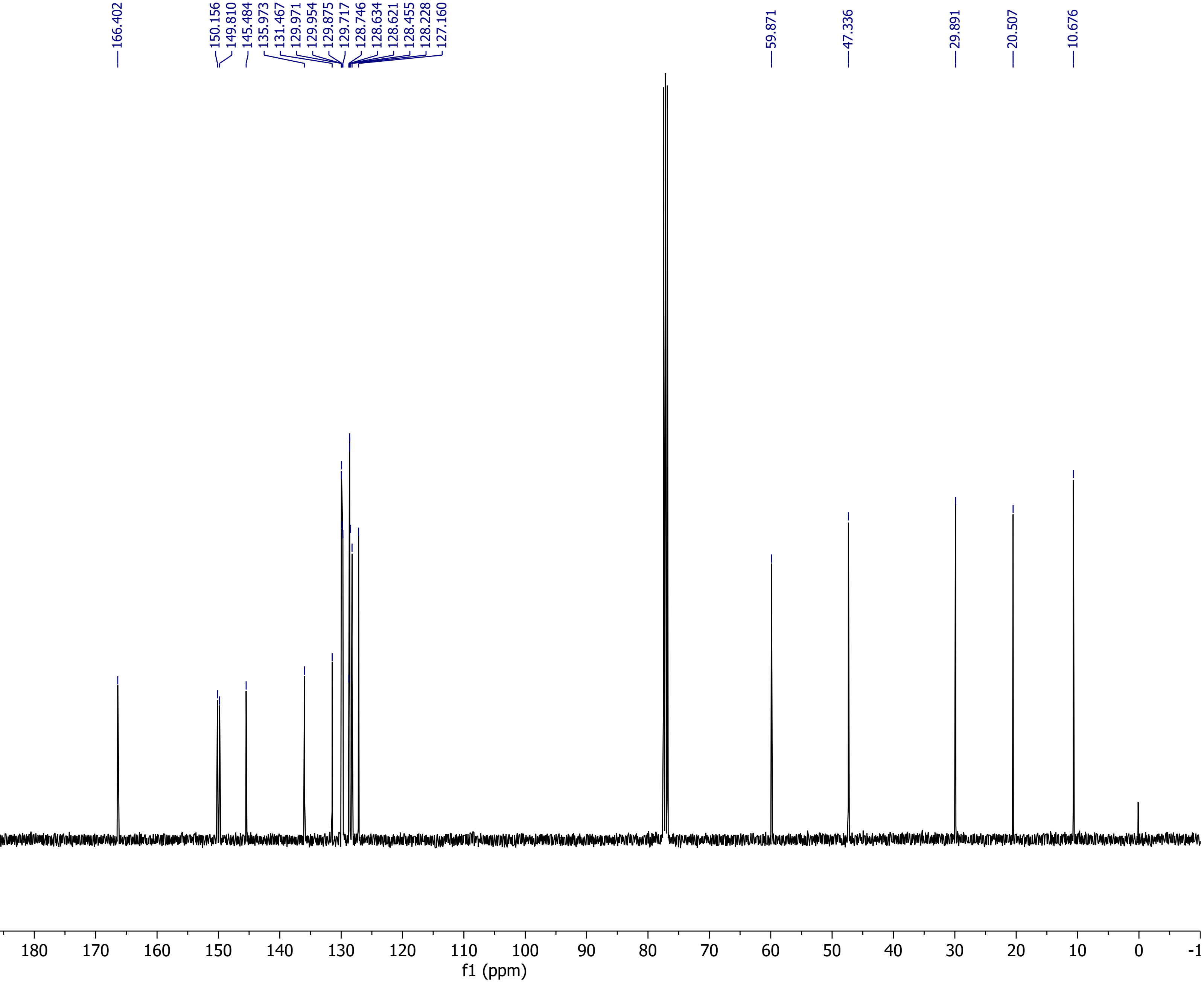

^{1}H NMR (400 MHz, CDCl$_3$)

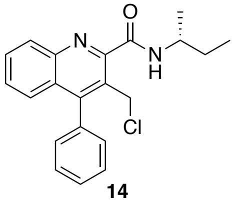

14

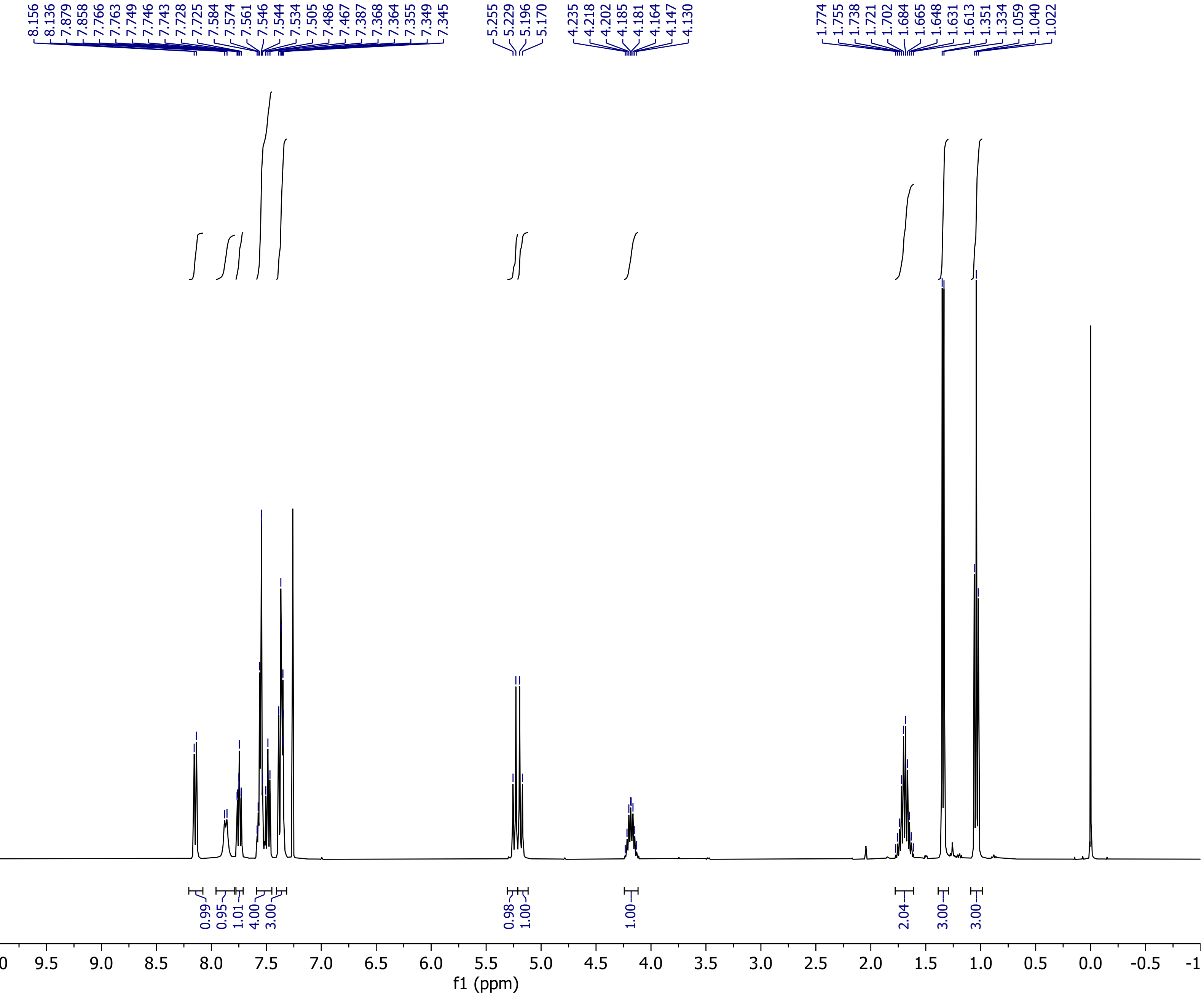

$^{13}C\{^1H\}$ NMR (101 MHz, $CDCl_3$)

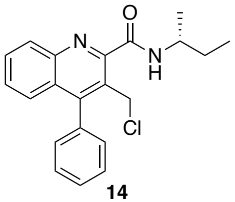

14

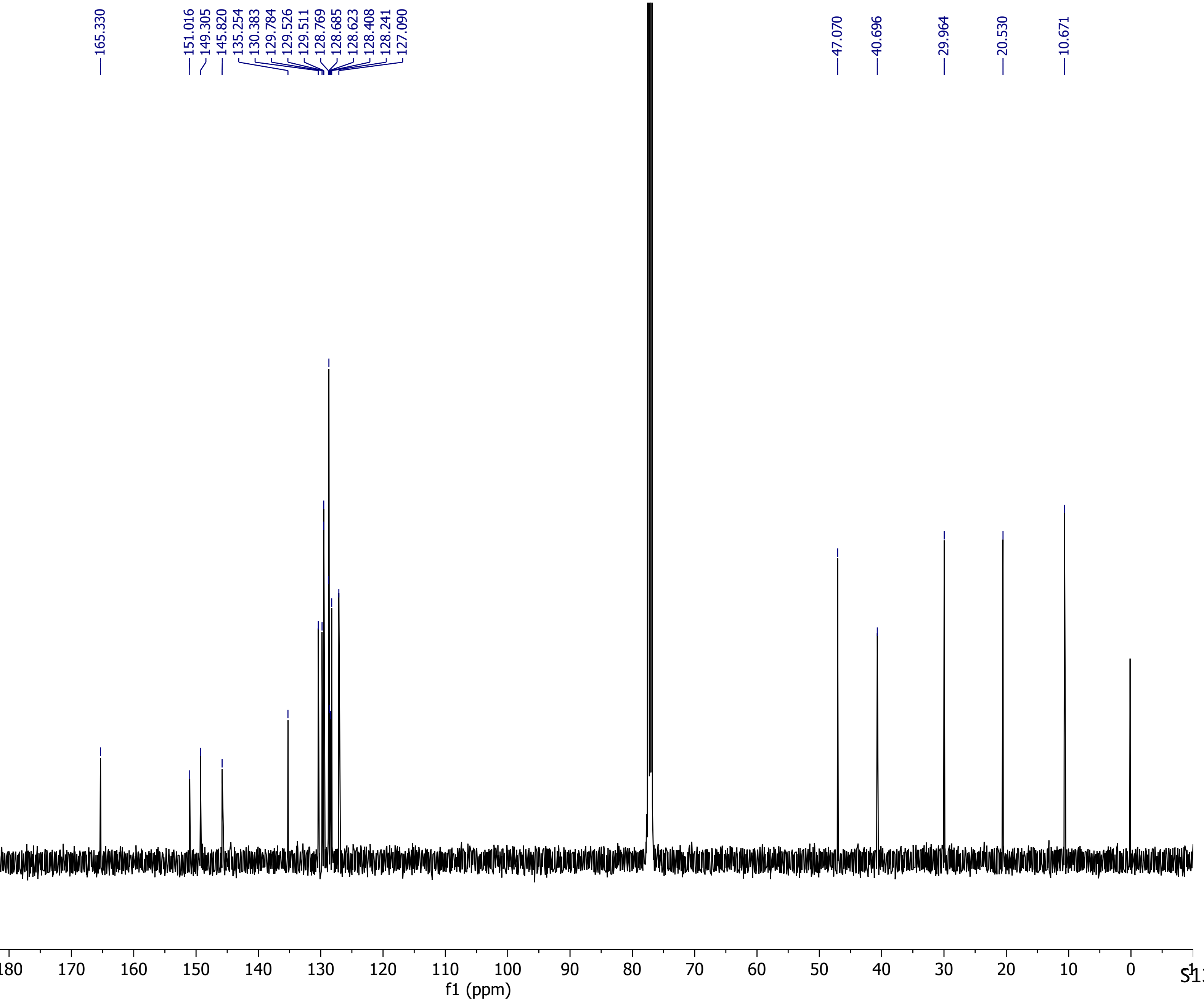

^{1}H NMR (400 MHz, $CDCl_3$)

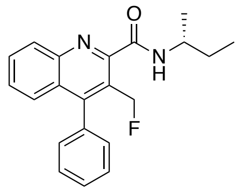

15

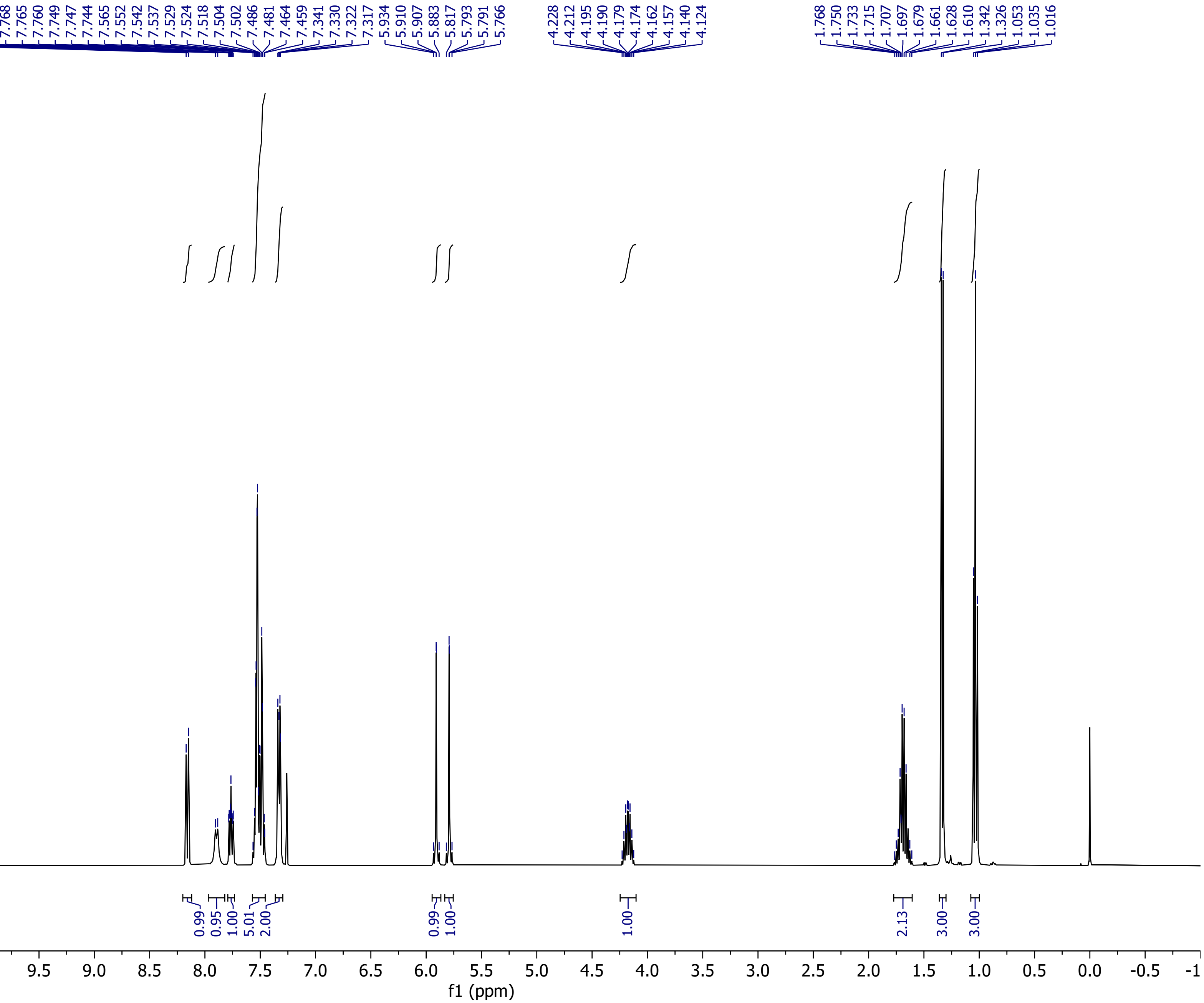

$^{13}C\{^{1}H\}$ NMR (101 MHz, CDCl$_3$)

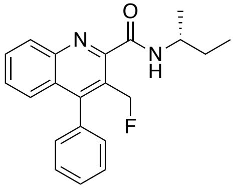

15

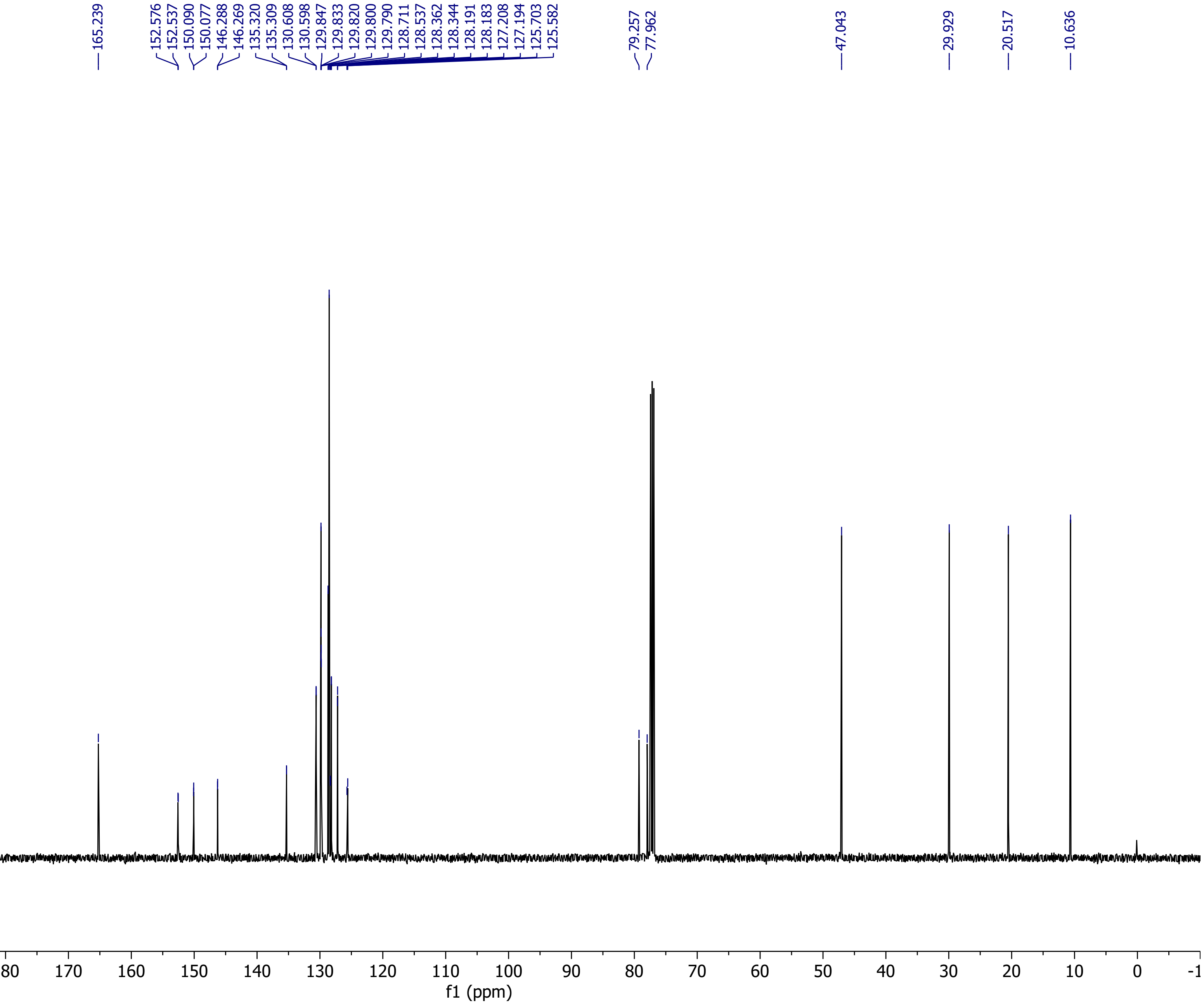

^{1}H NMR (400 MHz, $CDCl_3$)

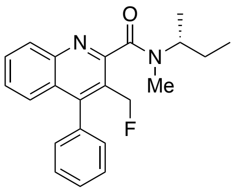

7

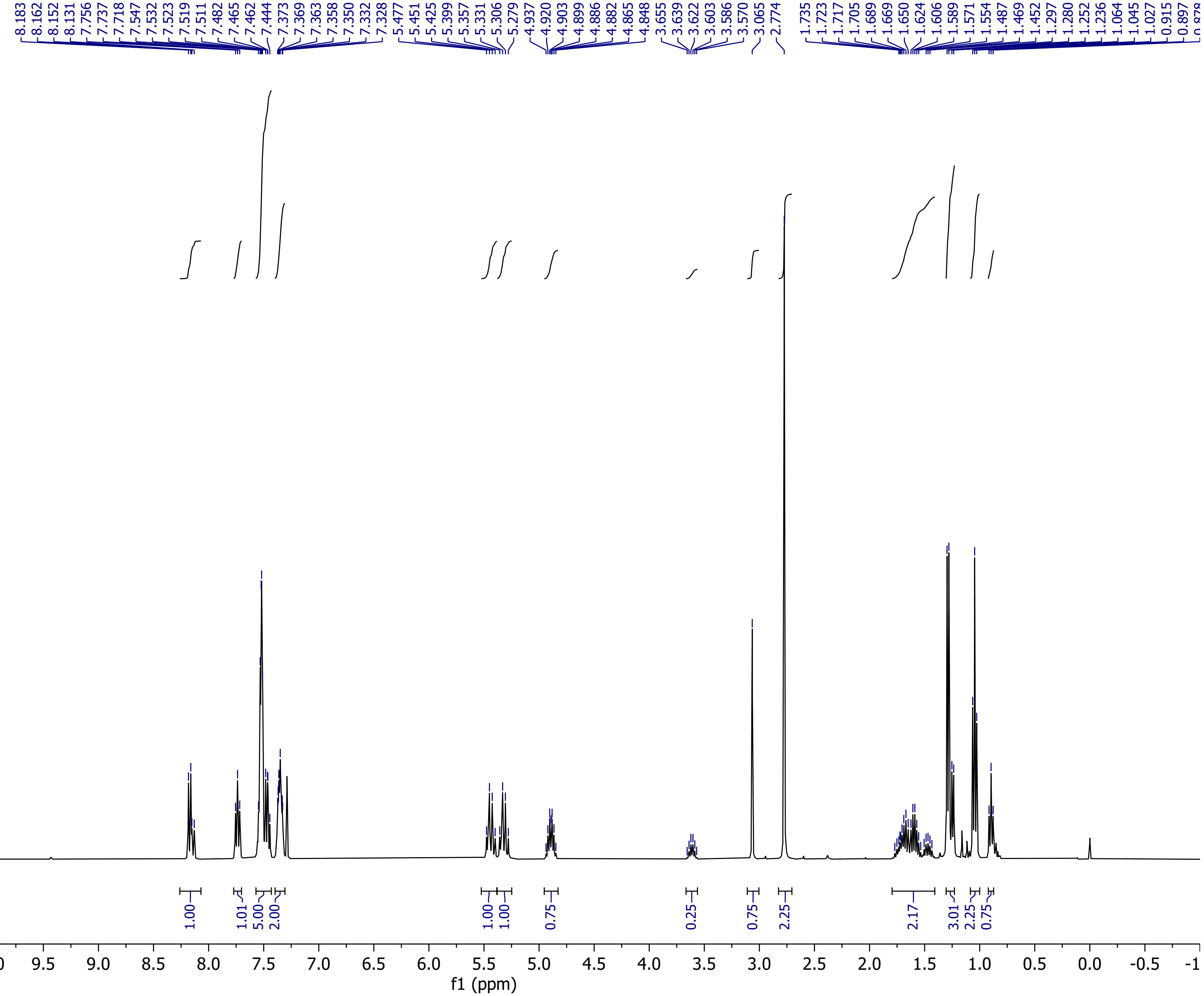

$^{13}C\{^{1}H\}$ NMR (101 MHz, $CDCl_3$)

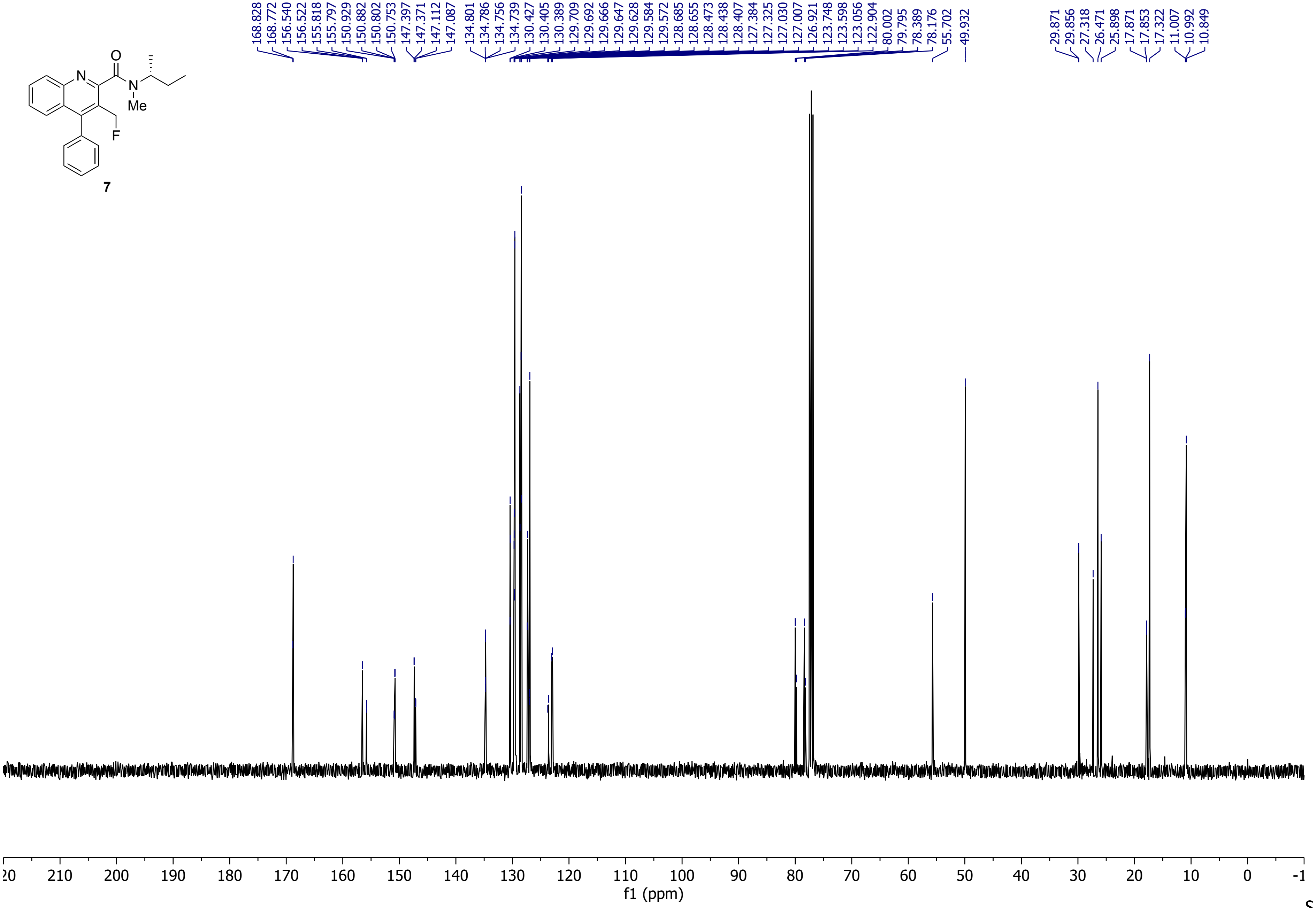

7

^{1}H NMR (400 MHz, $CDCl_3$)

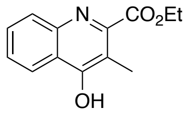

18

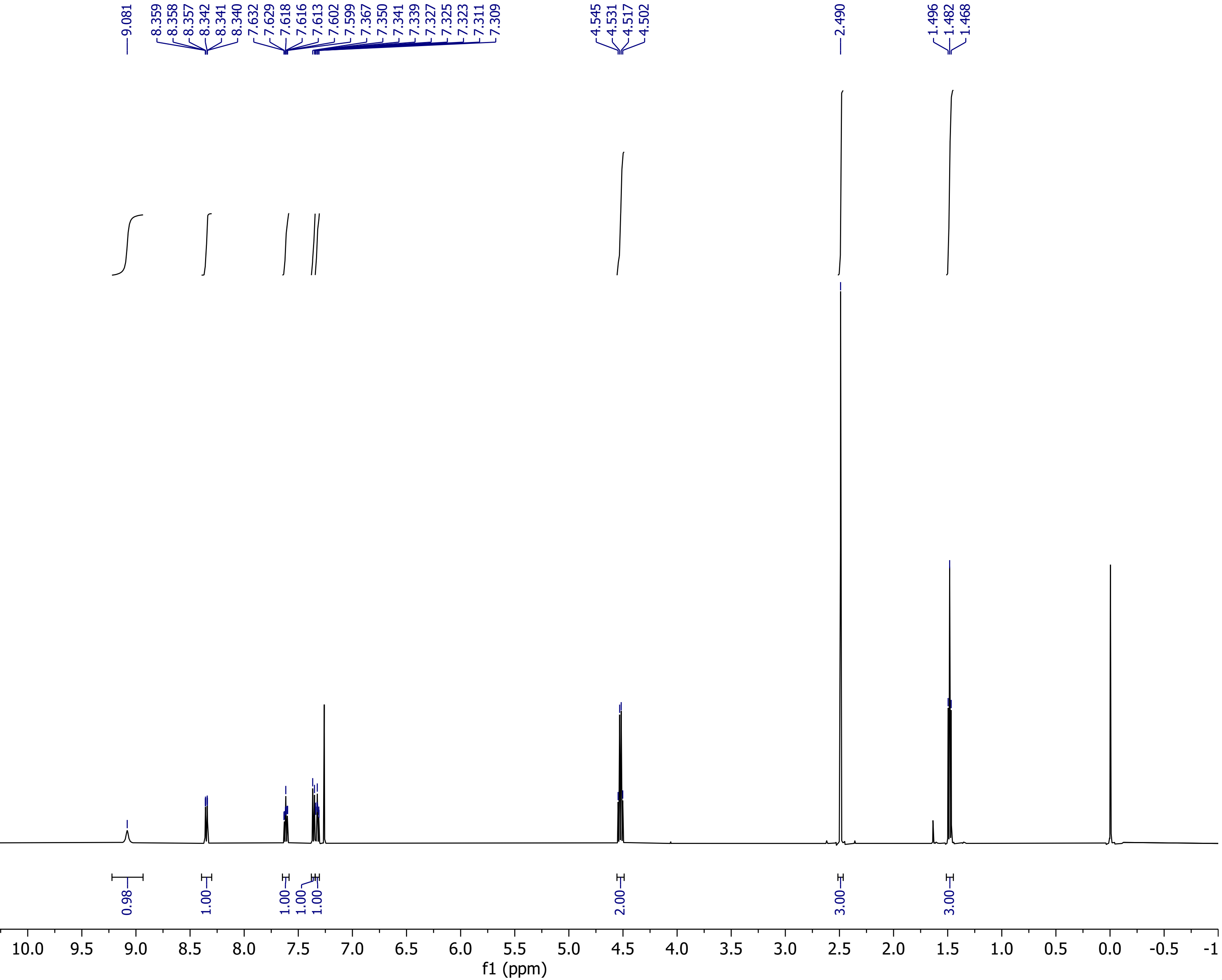

$^{13}C\{^1H\}$ NMR (101 MHz, $CDCl_3$)

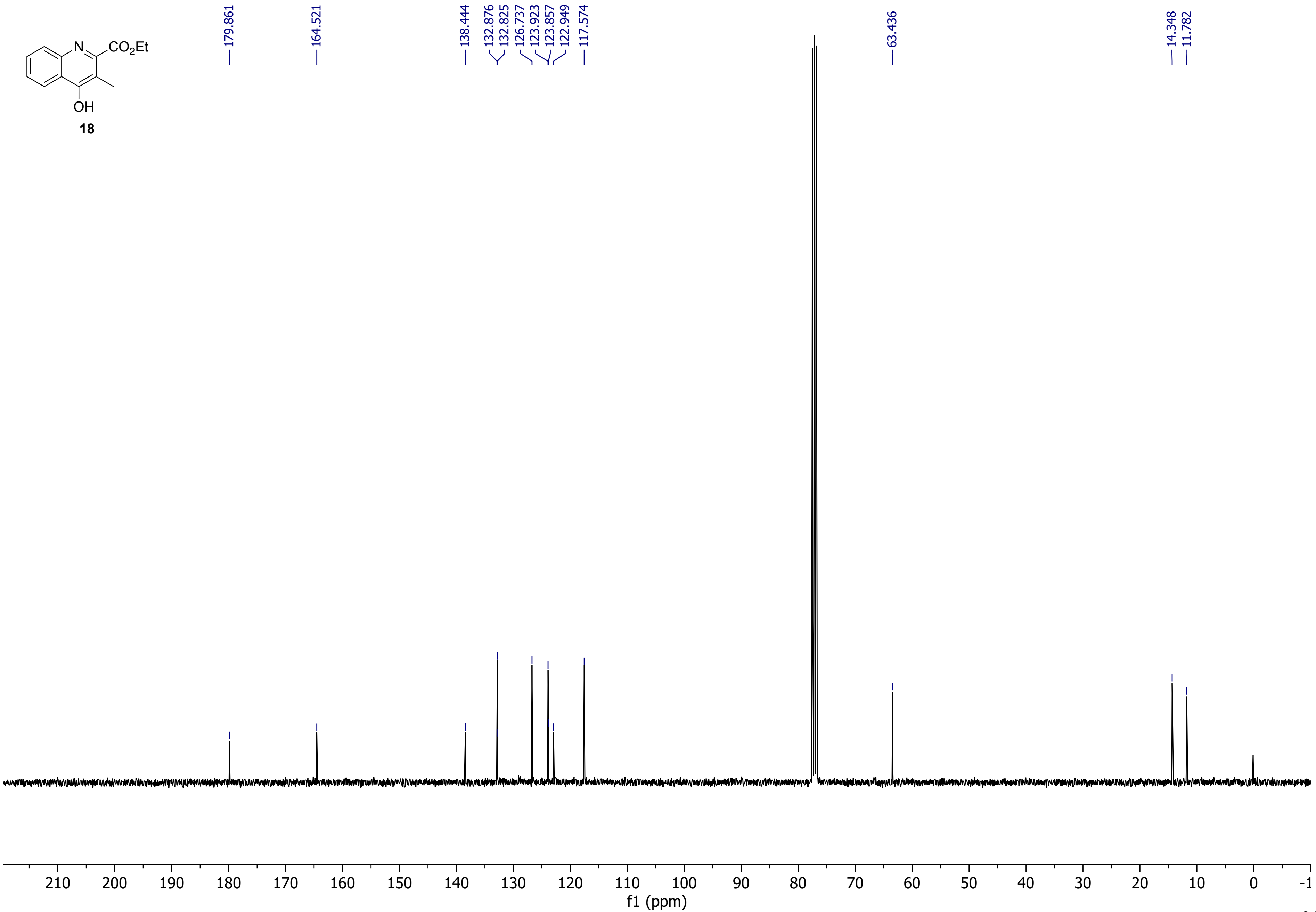

^{1}H NMR (400 MHz, $CDCl_3$)

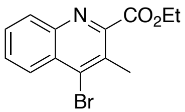

19

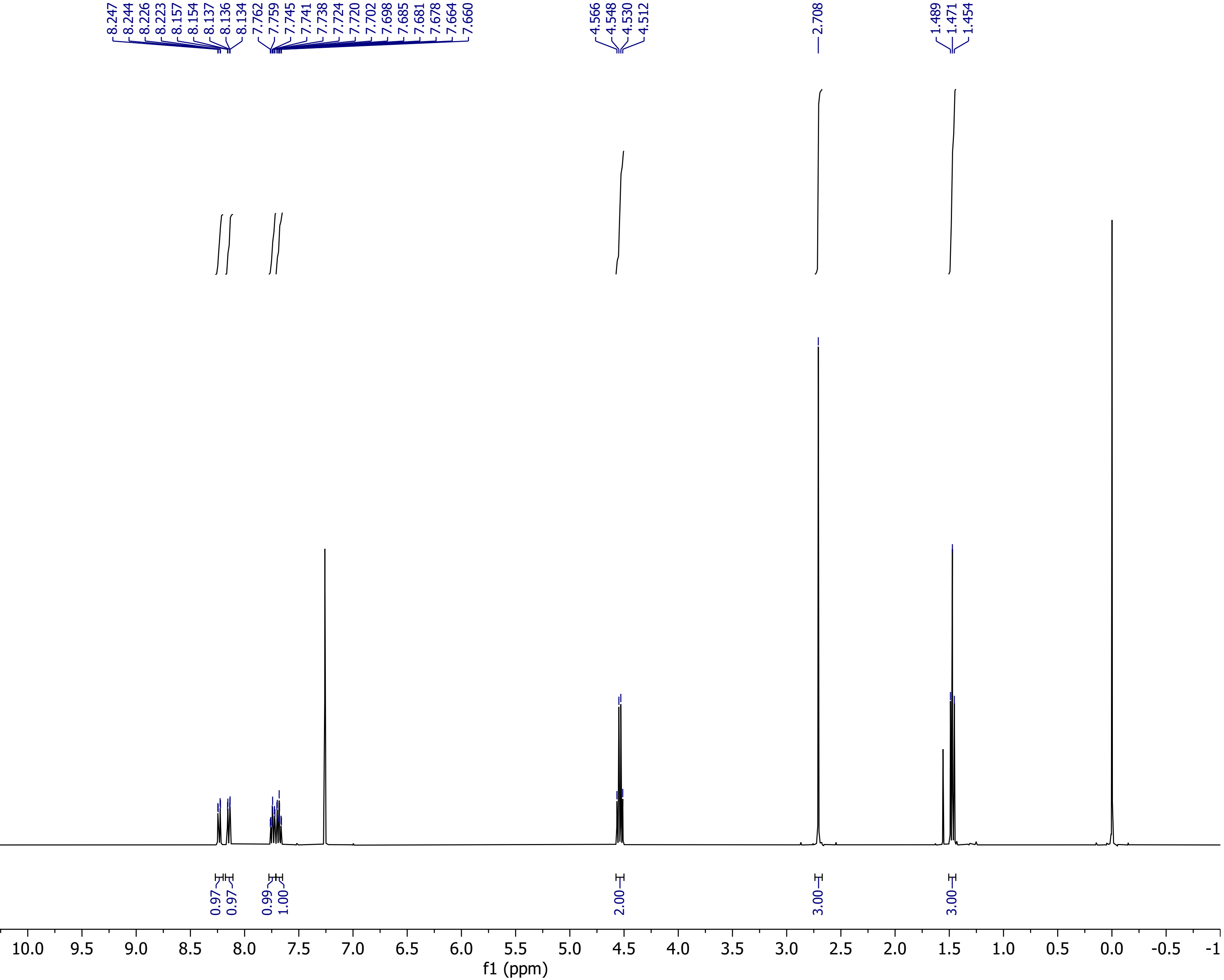

$^{13}C\{^{1}H\}$ NMR (101 MHz, $CDCl_3$)

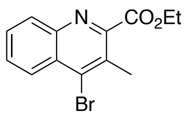

19

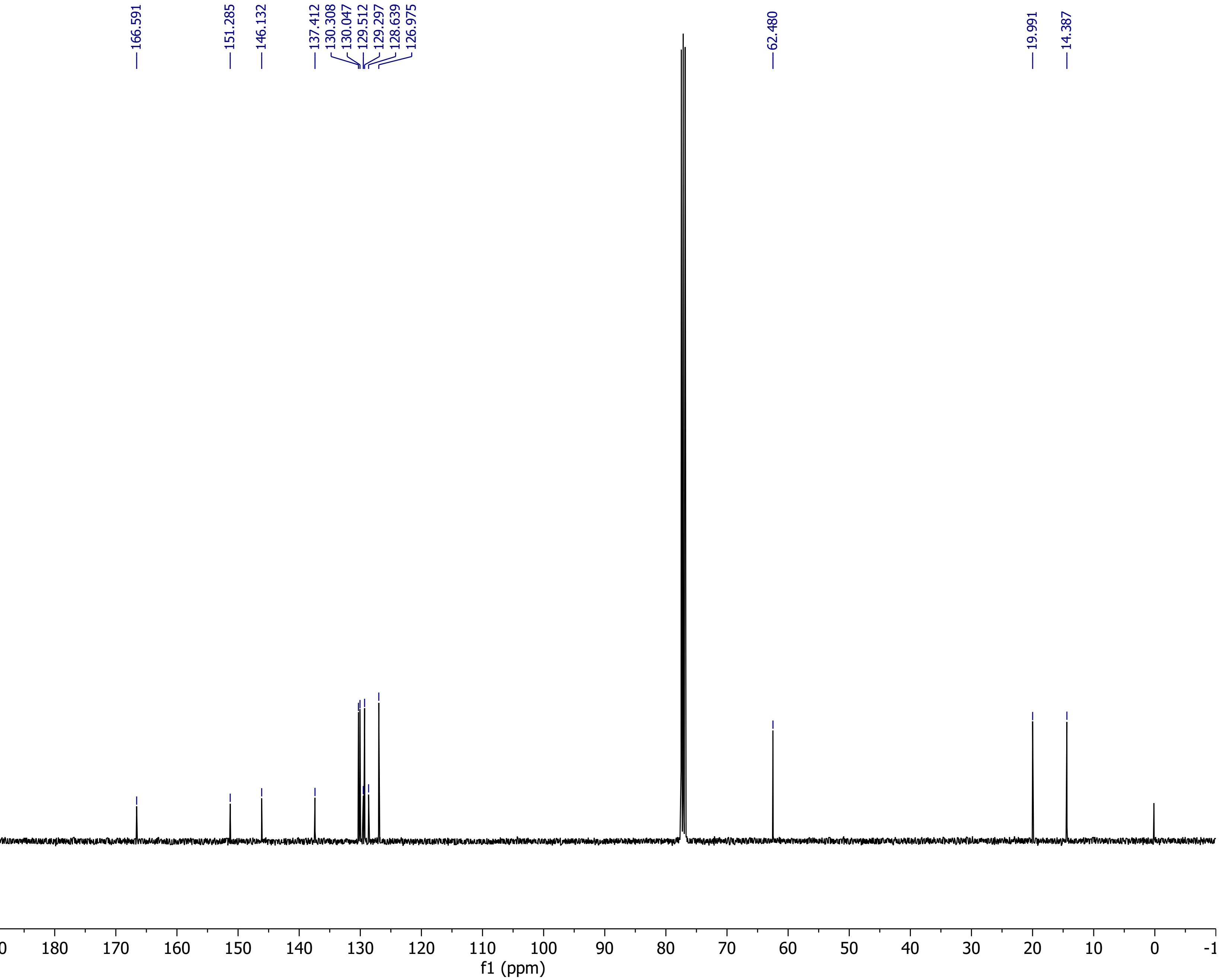

^{1}H NMR (400 MHz, $CDCl_3$)

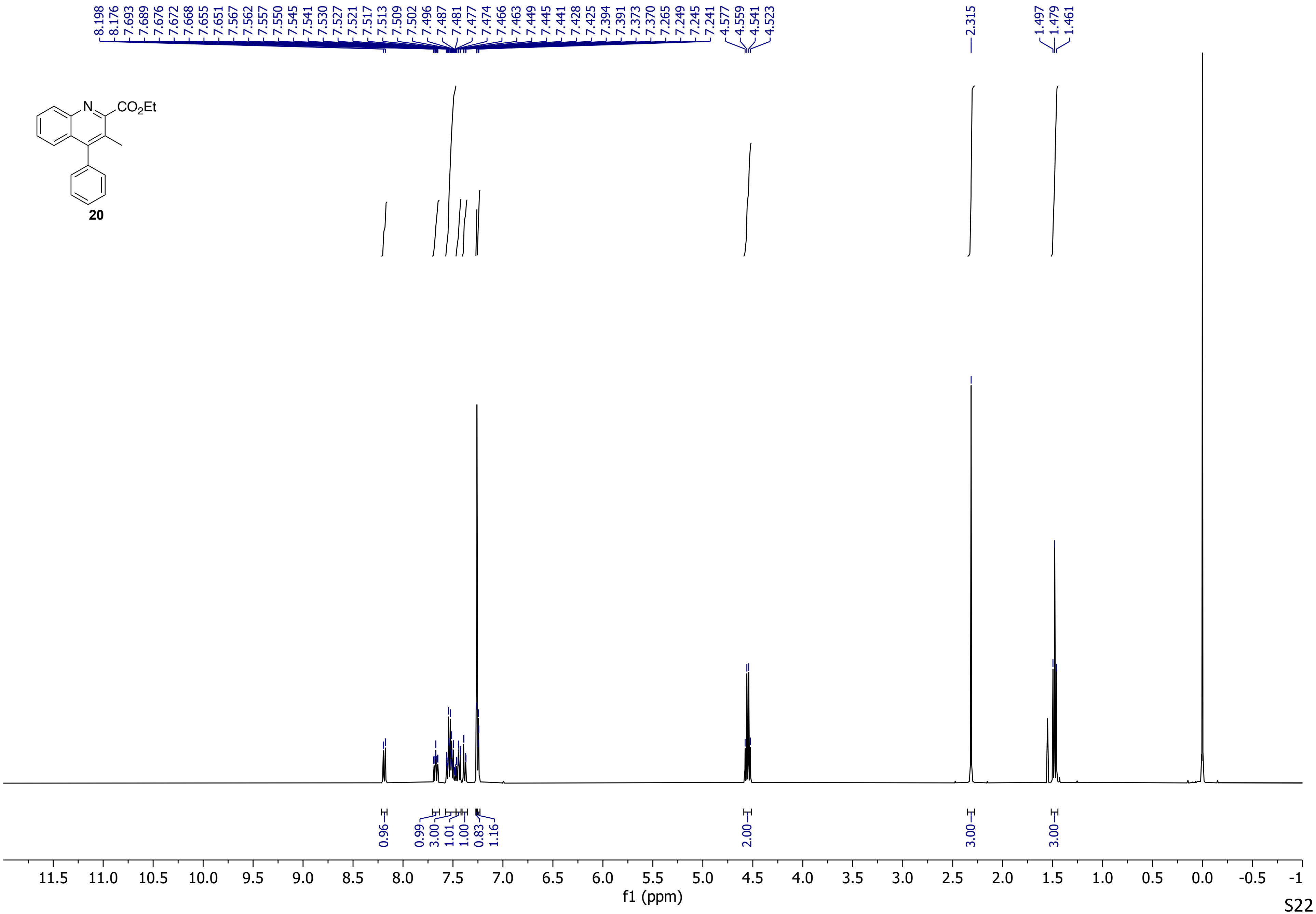

$^{13}C\{^{1}H\}$ NMR (101 MHz, $CDCl_3$)

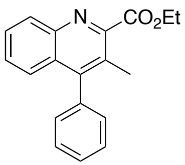

20

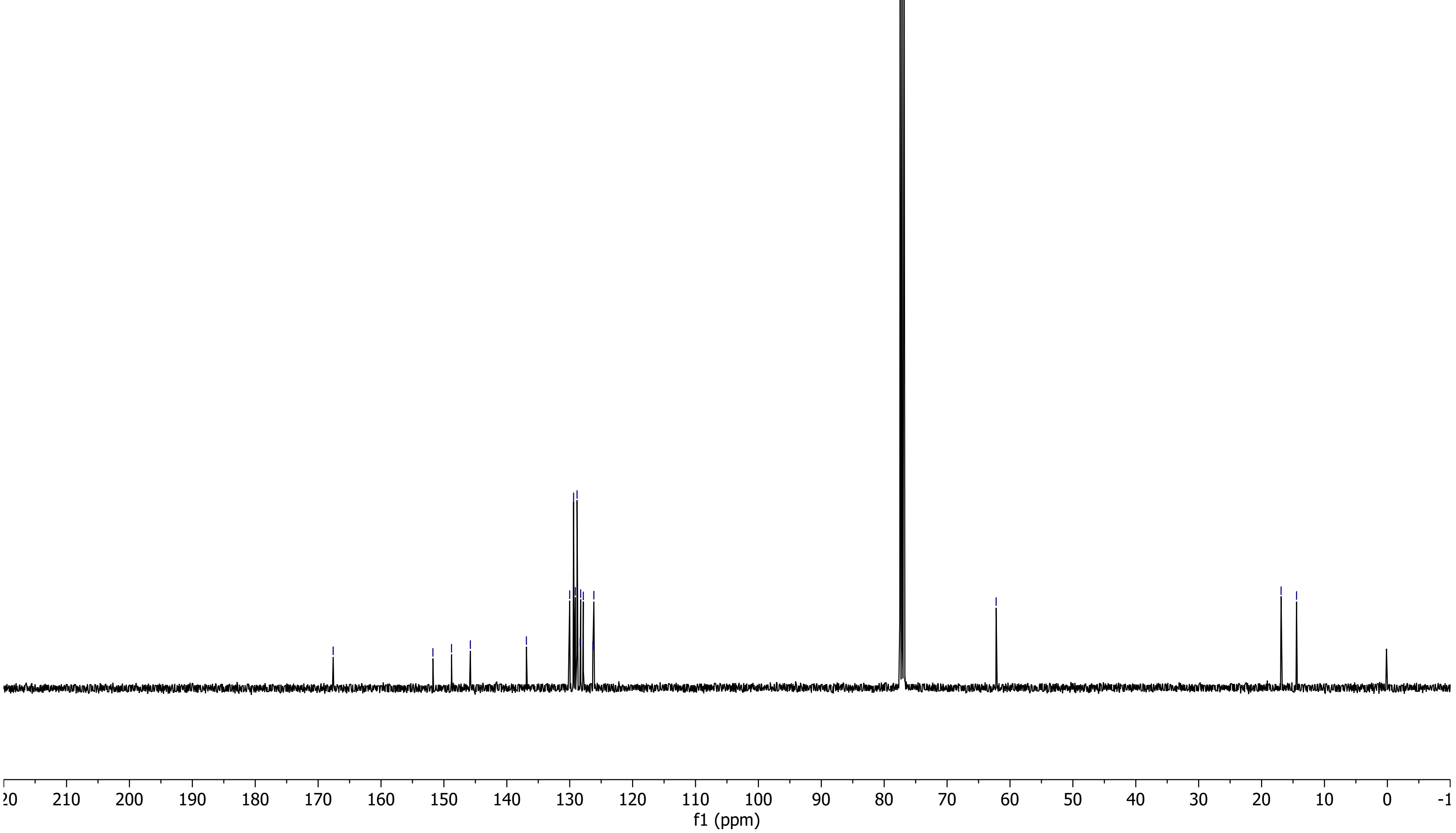

^{1}H NMR (400 MHz, $CDCl_3$)

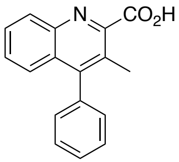

21

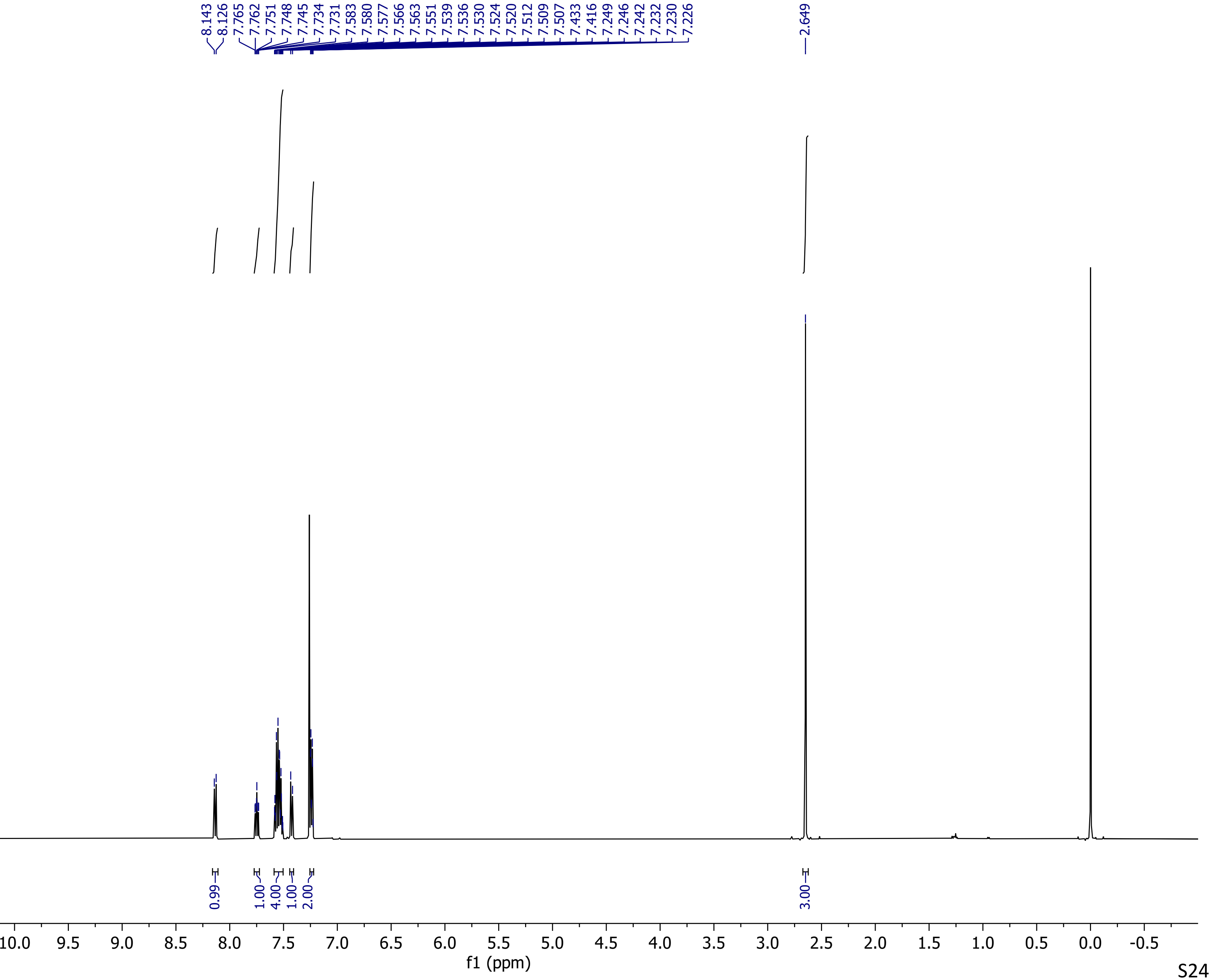

$^{13}C\{^1H\}$ NMR (101 MHz, $CDCl_3$)

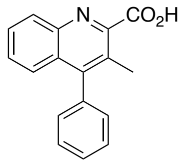

21

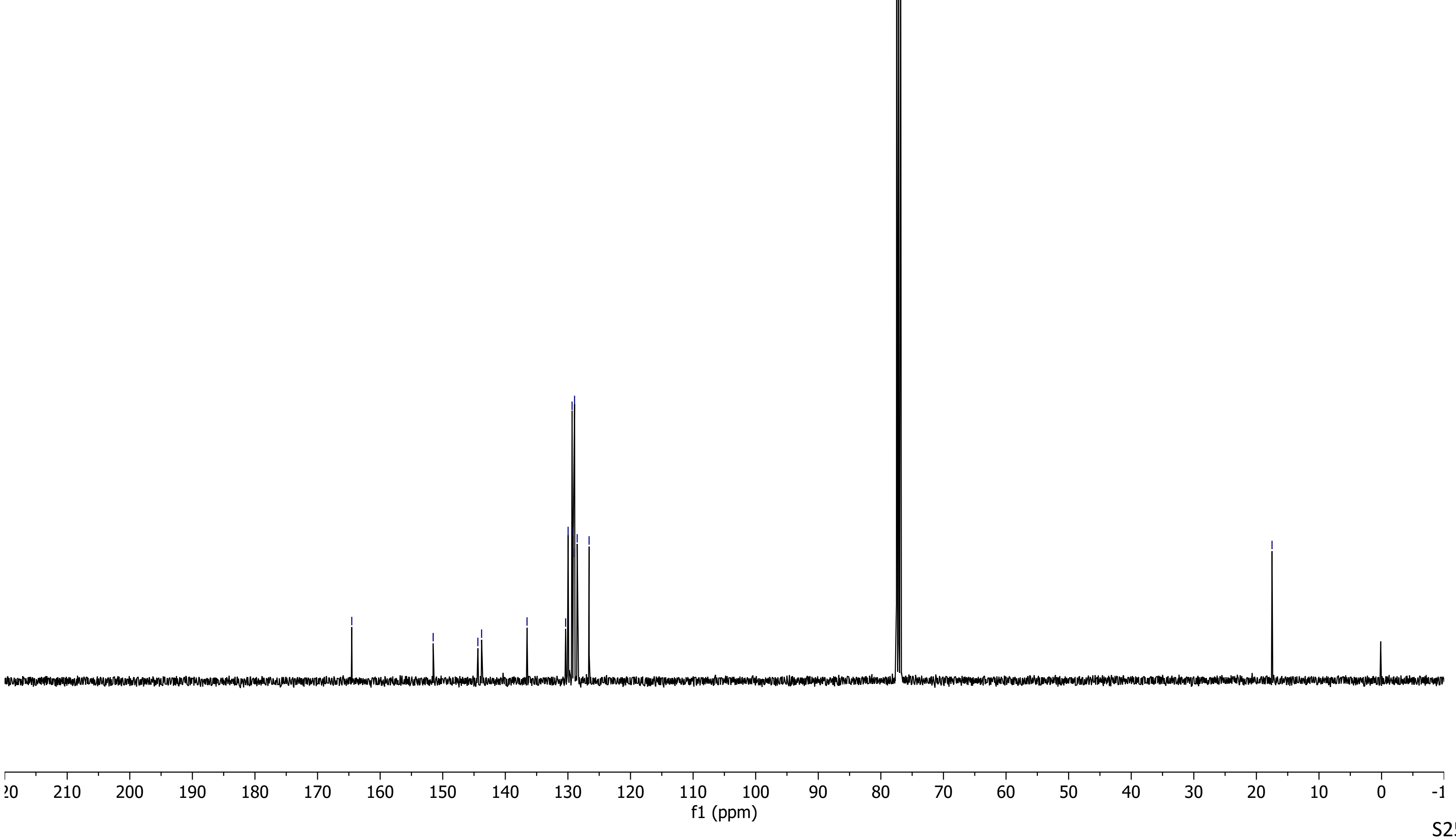

^{1}H NMR (400 MHz, $CDCl_3$)

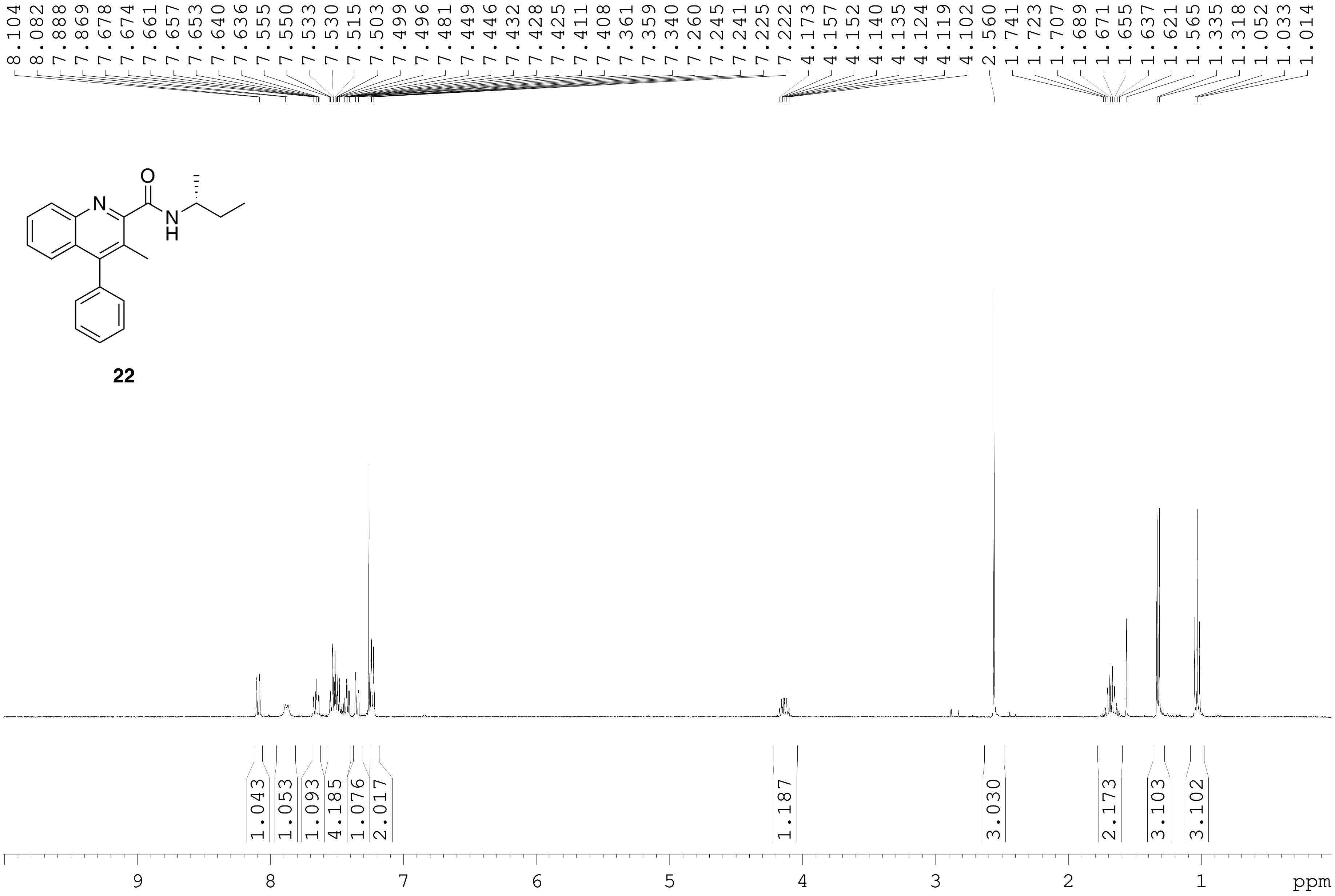

$^{13}C\{^1H\}$ NMR (101 MHz, $CDCl_3$)

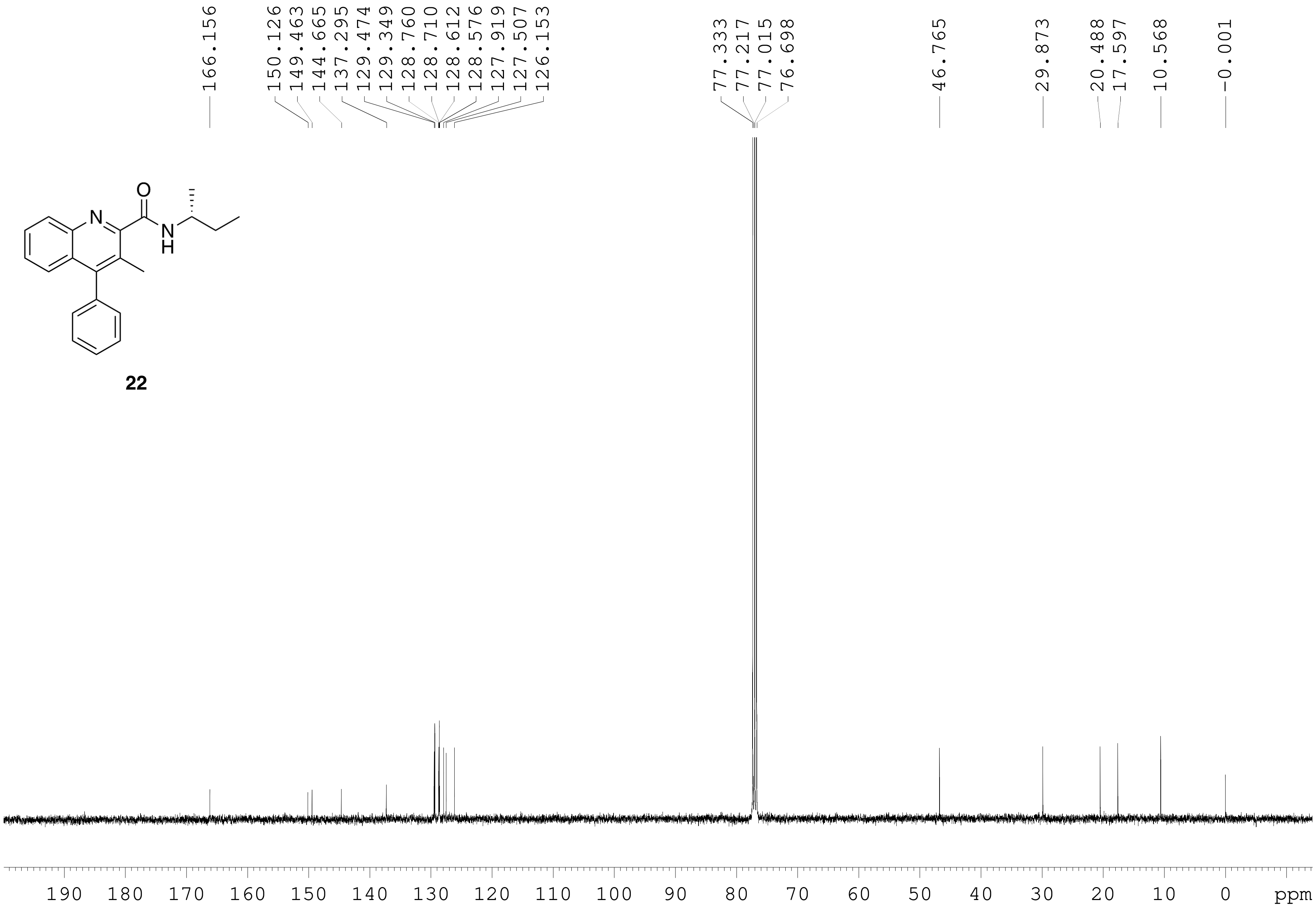

22

^{1}H NMR (400 MHz, $CDCl_3$)

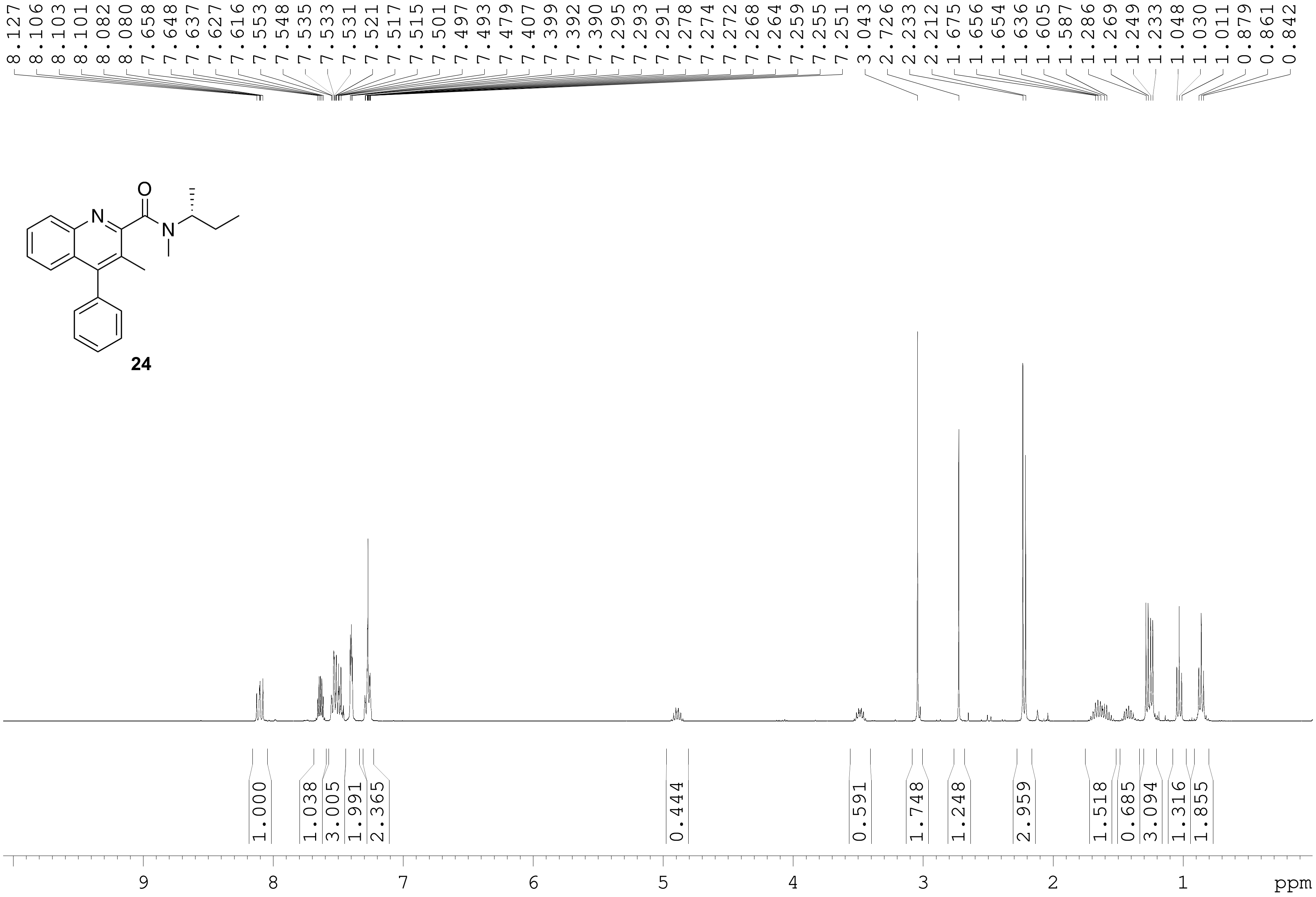

24

$^{13}C\{^{1}H\}$ NMR (101 MHz, CDCl$_3$)

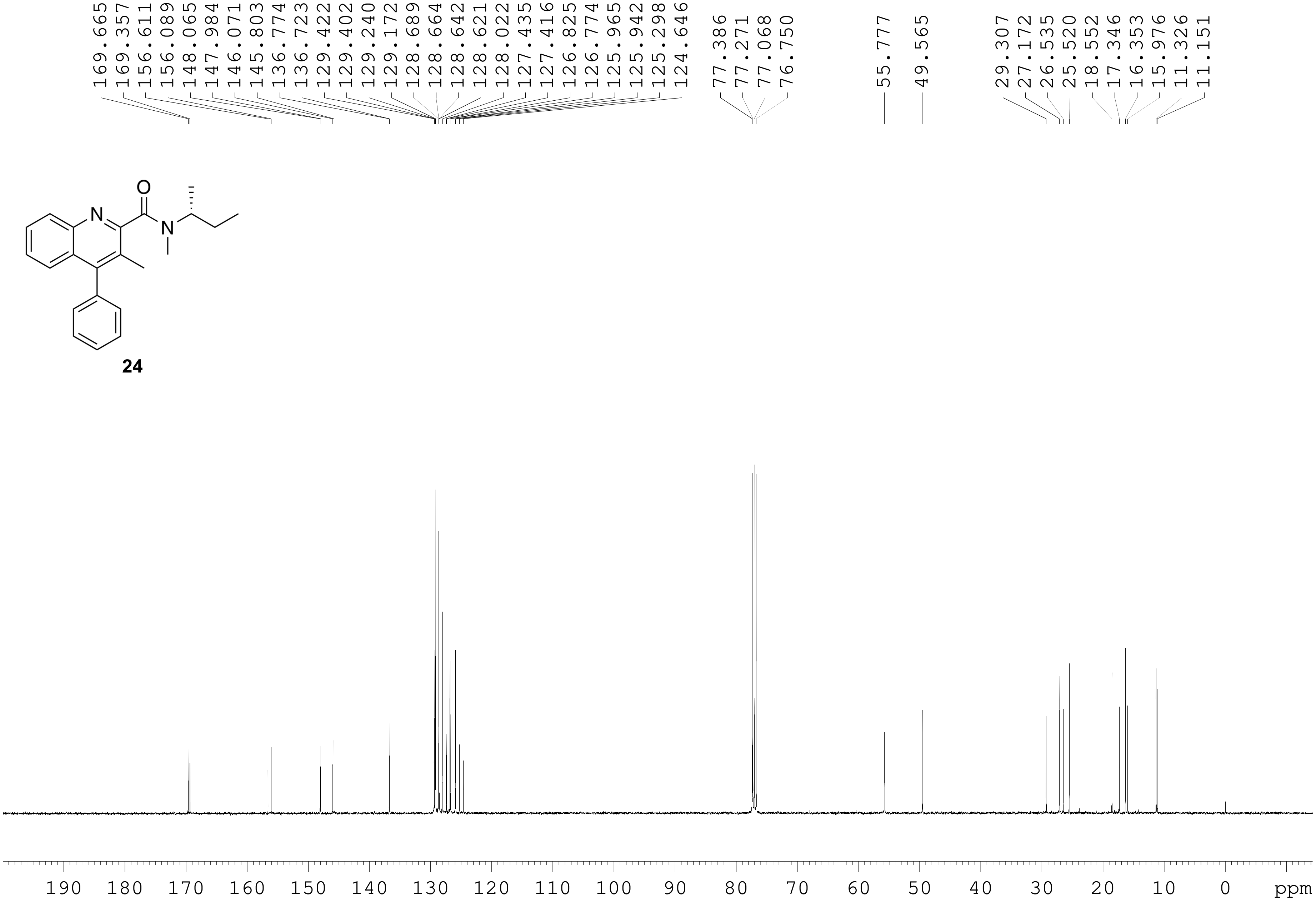

^{1}H NMR (400 MHz, $CDCl_3$)

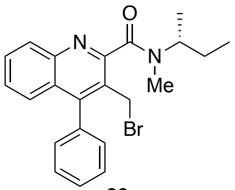

26

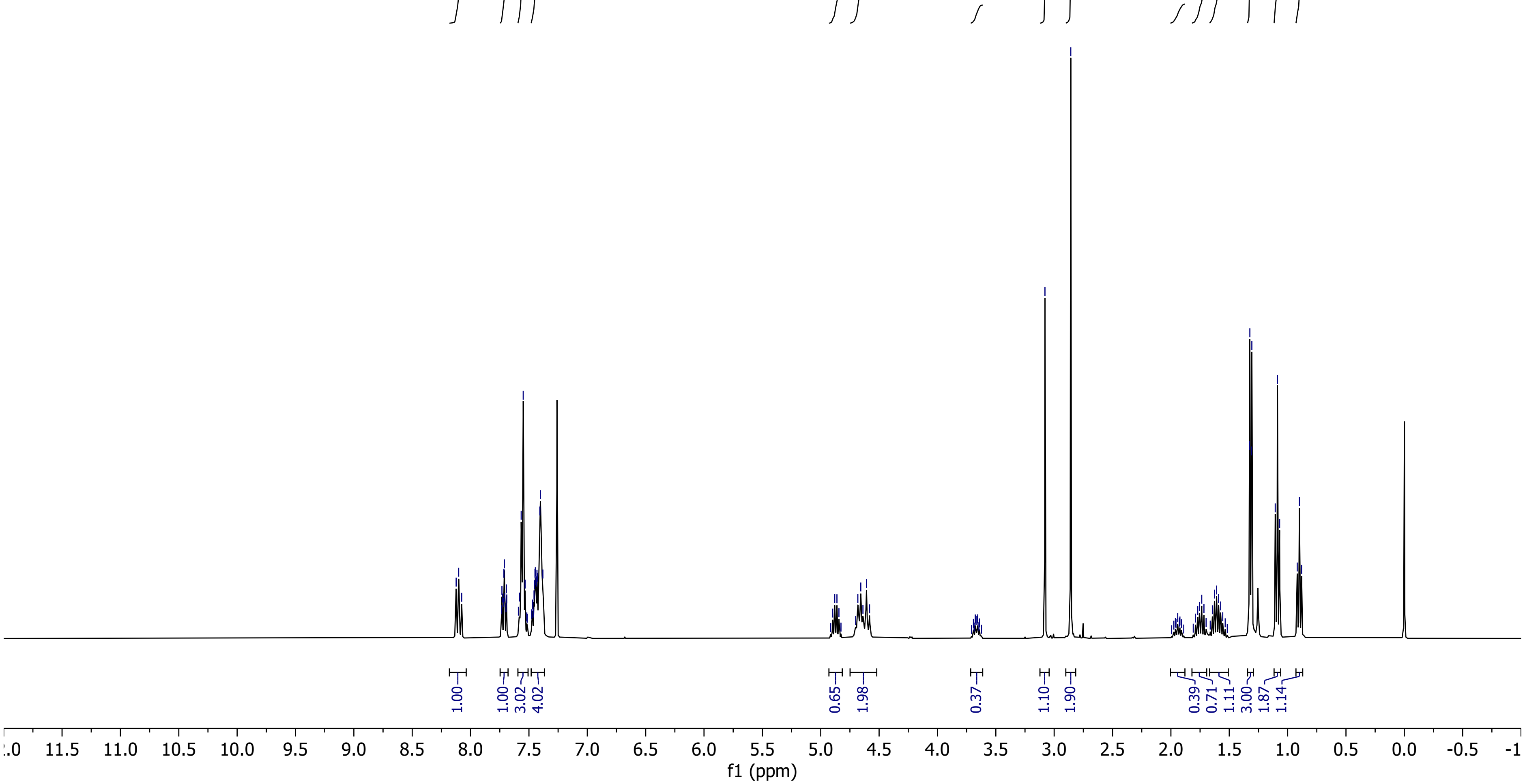

$^{13}C\{^1H\}$ NMR (101 MHz, $CDCl_3$)

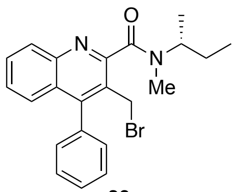

26

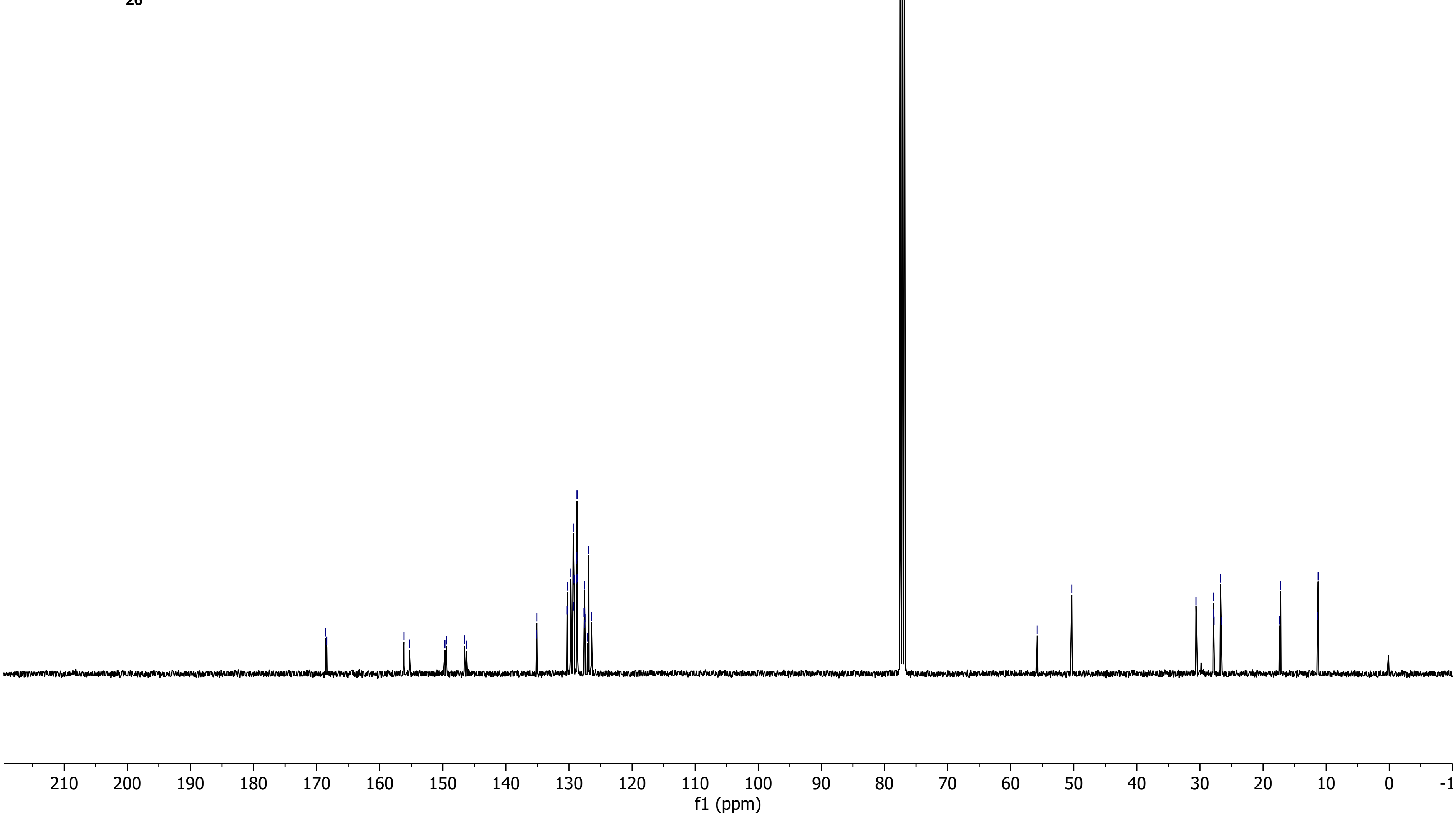

^{1}H NMR (400 MHz, $CDCl_3$)

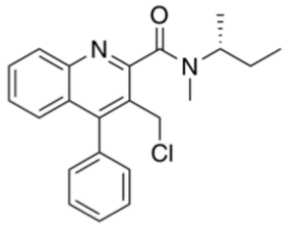

28

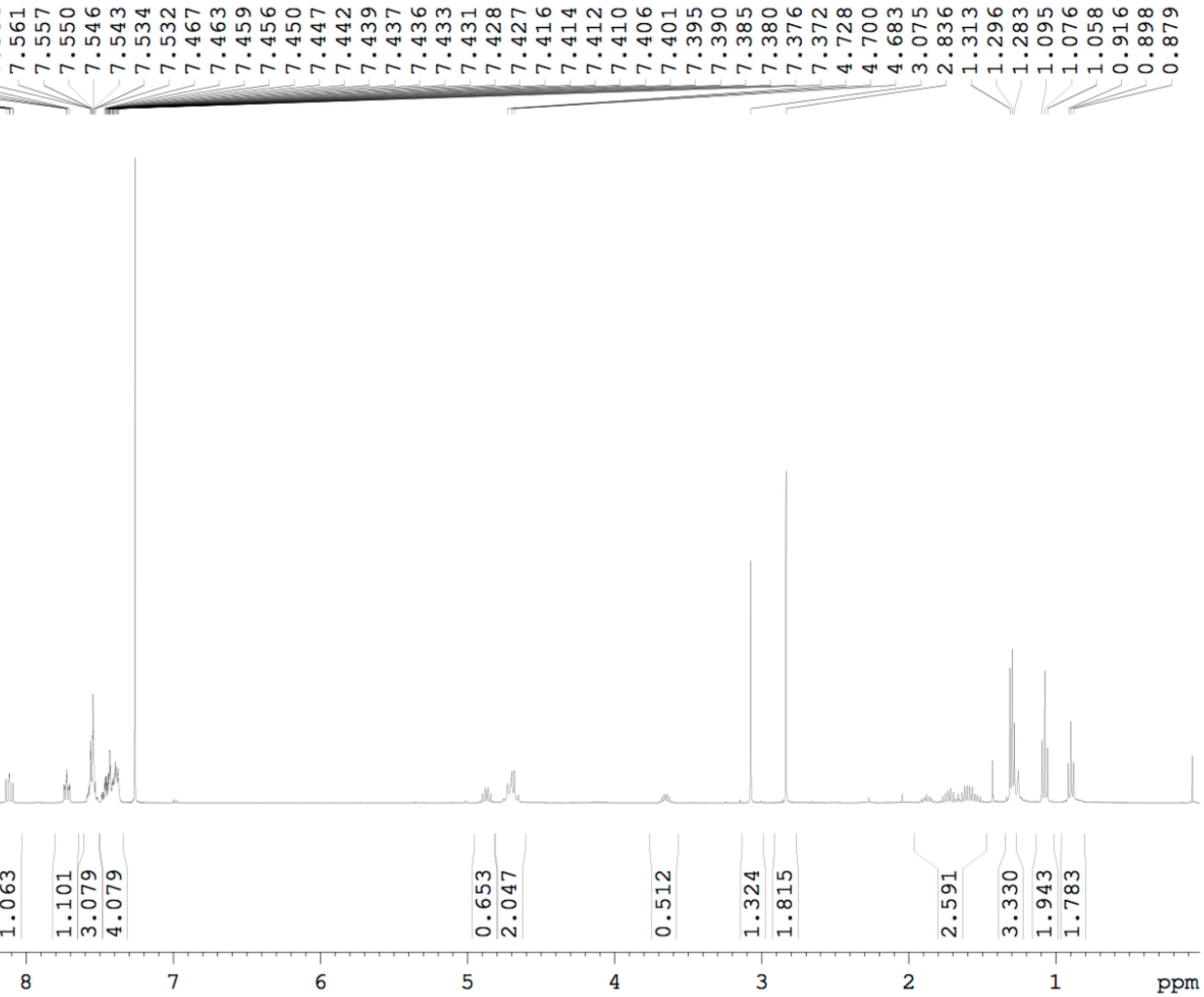

$^{13}C\{^{1}H\}$ NMR (101 MHz, $CDCl_3$)

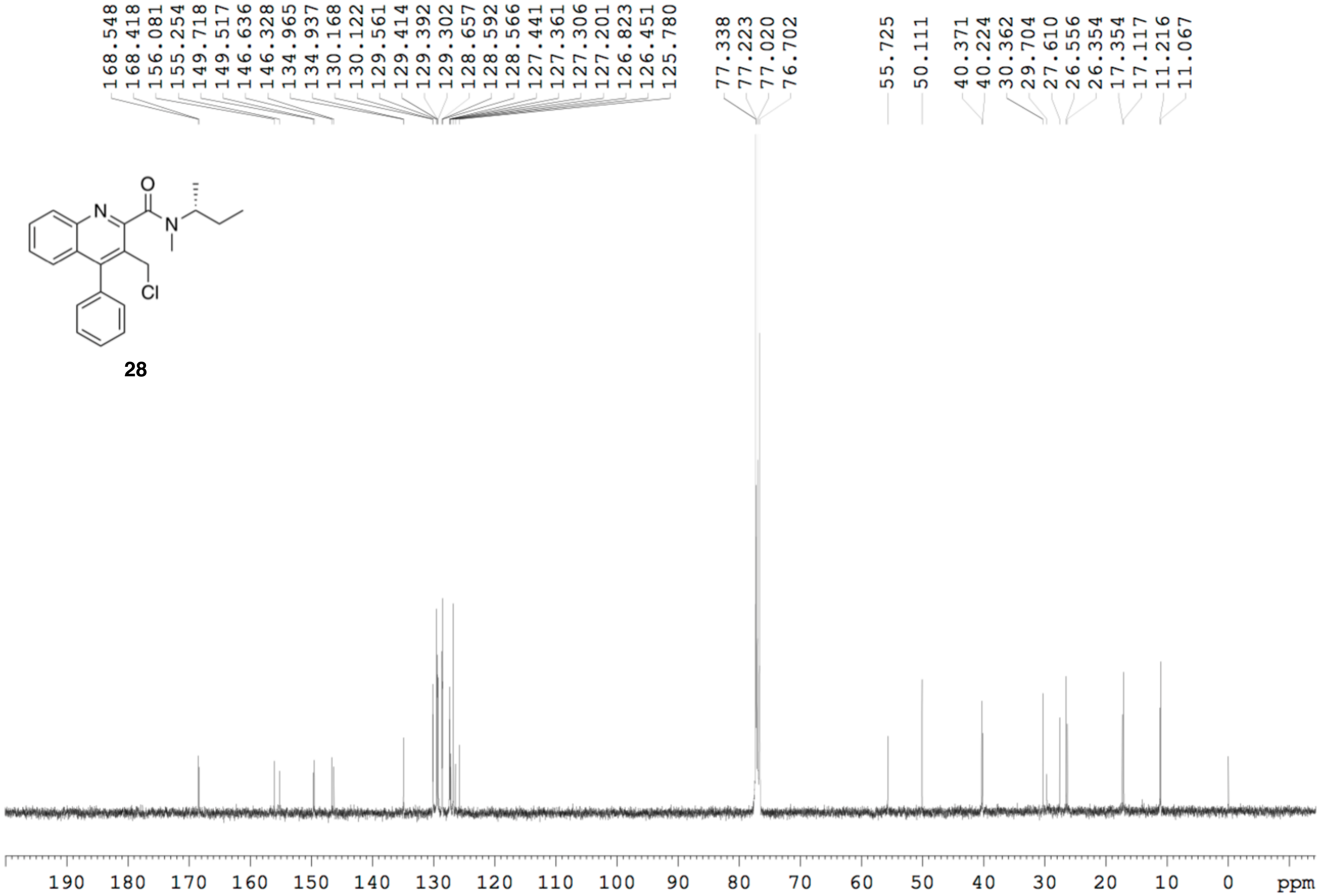

Supplement: MD-017-D5MD00930H-s001 [file MD-017-D5MD00930H-s001.pdf]
